# Complete trace models of state and control

**DOI:** 10.1007/978-3-030-72019-3_13

**Published:** 2021-03-23

**Authors:** Guilhem Jaber, Andrzej S. Murawski

**Affiliations:** Imperial College, London, UK; 9 Université de Nantes, LS2N CNRS, Inria, Nantes, France; 10 University of Oxford, Oxford, UK

**Keywords:** contextual equivalence, operational game semantics, higher-order references, control operators

## Abstract

We consider a hierarchy of four typed call-by-value languages with either higher-order or ground-type references and with either $\mathrm {call/cc}$ or no control operator.

Our first result is a fully abstract trace model for the most expressive setting, featuring both higher-order references and $\mathrm {call/cc}$, constructed in the spirit of operational game semantics. Next we examine the impact of suppressing higher-order references and callcc in contexts and provide an operational explanation for the game-semantic conditions known as visibility and bracketing respectively. This allows us to refine the original model to provide fully abstract trace models of interaction with contexts that need not use higher-order references or $\mathrm {call/cc}$. Along the way, we discuss the relationship between error- and termination-based contextual testing in each case, and relate the two to trace and complete trace equivalence respectively.

Overall, the paper provides a systematic development of operational game semantics for all four cases, which represent the state-based face of the so-called semantic cube.

### 1 Introduction

Research into contextual equivalence has a long tradition in programming language theory, due to its fundamental nature and applicability to numerous verification tasks, such as the correctness of compiler optimisations. Capturing contextual equivalence mathematically, i.e. the *full abstraction* problem [26], has been an important driving force in denotational semantics, which led, among others, to the development of game semantics [2,12]. Game semantics models computation through sequences of question- and answer-moves by two players, traditionally called O and P, who play the role of the context and the program respectively. Because of its interactive nature, it has often been referred to as a middle ground between denotational and operational semantics.

* The full version is available at https://hal.archives-ouvertes.fr/hal-03116698.

Over the last three decades the game-semantic approach has led to numerous fully abstract models for a whole spectrum of programming paradigms. Most papers in this strand follow a rather abstract pattern when presenting the models, emphasing structure and compositionality, often developing a correspondence with a categorical framework along the way to facilitate proofs. The operational intuitions behind the games are somewhat obscured in this presentation, and left to be discovered through a deeper exploration of proofs.

In contrast, *operational game semantics* aims to define models in which the interaction between the term and the environment is described through a carefully instrumented labelled transition system (LTS), built using the syntax and operational semantics of the relevant language. Here, the derived trace semantics can be shown to be fully abstract. In this line of work, the dynamics is described more directly and provides operational intuitions about the meaning of moves, while not immediately giving structural insights about the structure of the traces.

In this paper, we follow the operational approach and present a whole hierarchy of trace models for higher-order languages with varying access to higher-order state and control. As a vehicle for our study, we use HOSC, a call-by-value higher-order language equipped with general references and continuations. We also consider its sublanguages GOSC, HOS and GOS, obtained respectively by restricting storage to ground values, by removing continuations, and by imposing both restrictions. We study contextual testing of a class of HOSC terms using contexts from each of the languages $\mathbf{x} \in \{\mathrm{HOSC}, \mathrm{GOSC}, \mathrm{HOS}, \mathrm{GOS}\}$; we write $\mathbf{x}$ to refer to each case. Our working notion of convergence will be error reachability, where an error is represented by a free variable. Accordingly, at the technical level, we will study a family of equivalence relations $\cong^{\mathbf{x}}_{err}$, each corresponding to contextual testing with contexts from $\mathbf{x}$, where contexts have the extra power to abort the computation.

Our main results are trace models $\mathbf{Tr}_{\mathbf{x}}(\Gamma \vdash M)$ for each $\mathbf{x} \in \{\mathrm{HOSC}, \mathrm{GOSC}, \mathrm{HOS}, \mathrm{GOS}\}$, which capture $\cong^{\mathbf{x}}_{err}$ through trace equivalence:

$$\Gamma \vdash M_1 \cong^{\mathbf{x}}_{err} M_2 \text{ if and only if } \mathbf{Tr}_{\mathbf{x}}(\Gamma \vdash M_1) = \mathbf{Tr}_{\mathbf{x}}(\Gamma \vdash M_2).$$

It turns out that, for contexts with control (i.e. $\mathbf{x} \in \{\mathrm{HOSC}, \mathrm{GOSC}\}$), $\cong^{\mathbf{x}}_{err}$ coincides with the standard notion of contextual equivalence based on termination, written $\cong^{\mathbf{x}}_{ter}$. However, in the other two cases, the former is strictly more discriminating than the latter. We explain how to account for this difference in the trace-based setting, using *complete* traces.

A common theme that has emerged in game semantics is the comparative study of the power of contexts, as it turned out possible to identify combinatorial conditions, namely *visibility* [3] and *bracketing* [22], that correspond to contextual testing in the absence of general references and control constructs respectively. In brief, visibility states that not all moves can be played, but only those that are enabled by a "visible part" of the interaction, which could be thought of as functions currently in scope. Bracketing in turn imposes a discipline on answers, requiring that the topmost question be answered first. In the paper, we provide an operational reconstruction of both conditions.

$$
\begin{array}{lll}
\sigma,\tau & \triangleq & \mathrm{Unit} \mid \mathrm{Int} \mid \mathrm{Bool} \mid \mathrm{ref}\tau \mid \tau\times\sigma \mid \tau\to\sigma \mid \mathrm{cont}\ \tau \\
U,V & \triangleq & () \mid \mathbf{tt} \mid \mathbf{ff} \mid \widehat{n} \mid x \mid \ell \mid \langle U,V\rangle \mid \lambda x^{\tau}.M \mid \mathbf{rec}\ y(x^{\tau}).M \mid \mathrm{cont}_{\tau}\,K \\
M,N & \triangleq & V \mid \langle M,N\rangle \mid \pi_i M \mid MN \mid \mathrm{ref}_{\tau}\,M \mid !M \mid M:=N \mid \mathrm{if}\ M_1\ M_2\ M_3 \mid M\oplus N \\
 & & \mid M\boxdot N \mid M=N \mid \mathrm{call/cc}_{\tau}(x.M) \mid \mathrm{throw}_{\tau}\ \mathrm{M\ to}\ N \\
K & \triangleq & \bullet \mid \langle V,K\rangle \mid \langle K,M\rangle \mid \pi_i K \mid VK \mid KM \mid \mathrm{ref}_{\tau}\,K \mid !K \mid V:=K \mid K:=M \\
 & & \mid \mathrm{if}\ K\ M\ N \mid K\oplus M \mid V\oplus K \mid K\boxdot M \mid V\boxdot K \mid K=M \mid V=K \\
 & & \mid \mathrm{throw}_{\tau}\ \mathrm{V\ to}\ K \mid \mathrm{throw}_{\tau}\ \mathrm{K\ to}\ M \\
C & \triangleq & \bullet \mid \langle M,C\rangle \mid \langle C,M\rangle \mid \pi_i C \mid \lambda x^{\tau}.C \mid \mathbf{rec}\ y(x^{\tau}).C \mid MC \mid CM \mid \mathrm{ref}_{\tau}\,C \mid !C \\
 & & \mid C:=M \mid M:=C \mid \mathrm{if}\ C\ M\ N \mid \mathrm{if}\ M\ C\ N \mid \mathrm{if}\ M\ N\ C \mid C\oplus M \mid M\oplus C \\
 & & \mid C\boxdot M \mid M\boxdot C \mid C=M \mid M=C \mid \mathrm{call/cc}_{\tau}(x.C) \mid \mathrm{throw}_{\tau}\ \mathrm{C\ to}\ M \\
 & & \mid \mathrm{throw}_{\tau}\ \mathrm{M\ to}\ C
\end{array}
$$

Notational conventions: $x, y \in \mathbf{Var}$, $\ell \in \mathbf{Loc}$, $n \in \mathbb{Z}$, $i \in \{1,2\}$, $\oplus \in \{+,-,*\}$, $\boxdot \in \{=,<\}$
Syntactic sugar: $\mathrm{let}\, x = M \,\mathrm{in}\, N$ stands for $(\lambda x.N)M$ (if $x$ does not occur in $N$ we also write $M;N$)

**Fig. 1.** HOSC syntax

Overall, we propose a unifying framework for studying higher-order languages with state and control, which we hope will make the techniques of (operational) game semantics clearer to the wider community. The construction of the fully abstract LTSs is by no means automatic, as there is no general methodology for extracting trace semantics from game models. Some attempts in that direction have been reported in [25], but the type discipline discussed there is far too weak to be applied to the languages we study. As the most immediate precursor to our work, we see the trace model of contextual interactions between HOS contexts and HOS terms from [23]. In comparison, the models developed in this paper are more general, as they consider the interaction between HOSC terms and contexts drawn from any of the four languages ranged over by $\mathbf{x}$.

In the 1990s, Abramsky proposed a research programme, originally called the *semantic cube* [1], which concerned investigating extensions of the purely functional programming language PCF along various axes. From this angle, the present paper is an operational study of a *semantic diamond* of languages with state, with GOS at the bottom, extending towards HOSC at the top, either via GOSC or HOS.

### 2 HOSC

The main objects of our study will be the language HOSC along with its fragments GOSC, HOS and GOS. HOSC is a higher-order programming language equipped with general references and continuations.

*Syntax* HOSC syntax is given in Figure 1. Assuming countably infinite sets **Loc** (locations) and **Var** (variables), HOSC typing judgments take the form

$$
\begin{array}{ll|ll}
(K[(\lambda x^{\sigma}.M)V],h) & \rightarrow (K[M\{V/x\}],h) & (K[!\ell],h) & \rightarrow (K[h(\ell)],h) \\
(K[\pi_i\langle V_1,V_2\rangle],h) & \rightarrow (K[V_i],h) & (K[\mathrm{ref}\,V],h) & \rightarrow (K[\ell],h\cdot[\ell\mapsto V]) \\
(K[\mathrm{if}\ \mathbf{tt}\ M_1\ M_2],h) & \rightarrow (K[M_1],h) & (K[\ell:=V],h) & \rightarrow (K[()],h[\ell\mapsto V]) \\
(K[\mathrm{if}\ \mathbf{ff}\ M_1\ M_2],h) & \rightarrow (K[M_2],h) & (K[\ell=\ell'],h) & \rightarrow (K[b],h) \\
(K[\widehat{n}\oplus\widehat{m}],h) & \rightarrow (K[\widehat{n\oplus m}],h) & \multicolumn{2}{l}{\text{with } b=\mathbf{tt} \text{ if } \ell=\ell', \text{otherwise } b=\mathbf{ff}} \\
(K[\widehat{n}\boxdot\widehat{m}],h) & \rightarrow (K[b],h) & \multicolumn{2}{l}{(K[(\underbrace{\mathbf{rec}\ y(x^{\sigma}).M}_{U})V],h)} \\
\multicolumn{2}{l|}{\text{with } b=\mathbf{tt} \text{ if } n\boxdot m, \text{otherwise } b=\mathbf{ff}} & & \rightarrow (K[M\{V/x,U/y\}],h) \\
(K[\mathrm{call/cc}(x.M)],h) & \rightarrow (K[M\{\mathrm{cont}\,K/x\}],h) & (K[\mathrm{throw}\ \mathrm{V}\ \mathrm{to}\ \mathrm{cont}\,K'],h) & \rightarrow (K'[V],h)
\end{array}
$$

**Fig. 2.** Operational reduction for HOSC

$\Sigma;\Gamma \vdash M : \tau$, where $\Sigma$ and $\Gamma$ are finite partial functions that assign types to locations and variables respectively. In typing judgements, we often write $\Sigma$ as shorthand for $\Sigma;\emptyset$ (closed) and $\Gamma$ as shorthand for $\emptyset;\Gamma$ (location-free). Similarly, $\vdash M : \tau$ means $\emptyset;\emptyset \vdash M : \tau$.

*Operational semantics* A heap $h$ is a finite type-respecting map from **Loc** to values. We write $h : (\Sigma;\Gamma)$, if $\mathrm{dom}(\Sigma) \subseteq \mathrm{dom}(h)$ and $\Sigma;\Gamma \vdash h(\ell) : \sigma$ for $(\ell,\sigma) \in \Sigma$, The operational semantics of HOSC reduces pairs $(M,h)$, where $\Sigma;\Gamma \vdash M : \tau$ and $h : (\Sigma;\Gamma)$. The rules are given in Figure 2, where $\{\cdot\}$ denotes (capture-avoiding) substitution. We write $(M,h) \Downarrow_{ter}$ if there exist $V, h'$ such that $(M,h) \rightarrow^* (V,h')$ and $V$ is a value.

We distinguish the following fragments of HOSC.

**Definition 1.** – GOSC *types are* HOSC *types except that reference types are restricted to* $\mathrm{ref}\,\iota$, *where* $\iota$ *is given by the grammar* $\iota \triangleq \mathrm{Unit} \mid \mathrm{Int} \mid \mathrm{Bool} \mid \mathrm{ref}\,\iota$. GOSC *terms are* HOSC *terms whose typing derivations (i.e. not only the final typing judgments) rely on* GOSC *types only.* GOSC *is a superset of* FOSC *[8] (*GOSC *also includes references to references - the* $\mathrm{ref}\,\iota$ *case above).*

- HOS *types are* HOSC *types that do not feature the* cont *constructor.* HOS *terms are* HOSC *terms whose typing derivations rely on* HOS *types only. Consequently,* HOS *terms never have subterms of the form* $\mathrm{call/cc}_{\tau}(x.M)$, $\mathrm{throw}_{\tau}$ M to $N$ *or* $\mathrm{cont}_{\tau}\,K$.
- GOS *is the intersection of* HOS *and* GOSC*, both for types and terms, i.e. there are no continuations and storage is restricted to values of type* $\iota$*, defined above.*

**Definition 2.** *Given a* HOSC *term* $\Gamma \vdash M : \tau$*, we refer to types in* $\Gamma$ *and* $\tau$ *as* **boundary types**. *Let* $\mathbf{x} \in \{\mathrm{HOSC}, \mathrm{GOSC}, \mathrm{HOS}, \mathrm{GOS}\}$*. We say that a* HOSC *term* $\Gamma \vdash M : \tau$ *has an* $\mathbf{x}$ *boundary if all of its boundary types are from* $\mathbf{x}$.

*Remark 1.* Note that typing derivations of HOSC terms with an $\mathbf{x}$ boundary may contain arbitrary HOSC types as long as the final typing judgment uses types from $\mathbf{x}$ only. Consequently, if $\mathbf{x} \neq$ HOSC, HOSC terms with an $\mathbf{x}$ boundary form a strict superset of $\mathbf{x}$.

Next we introduce several notions of contextual testing for HOSC-terms, using various kinds of contexts. For a start, we introduce the classic notion of contextual approximation based on observing termination. The notions are parameterized by $\mathbf{x}$, indicating which language is used to build the testing contexts. We write $\Gamma \vdash C : \tau \to \tau'$ if $\Gamma, x : \tau \vdash C[x] : \tau'$, and $\Gamma \vdash C \div \tau$ if $\Gamma \vdash C : \tau \to \tau'$ for some $\tau'$.

**Definition 3 (Contextual Approximation).** *Let* $\mathbf{x} \in \{\mathrm{HOSC}, \mathrm{GOSC}, \mathrm{HOS}, \mathrm{GOS}\}$*. Given* HOSC *terms* $\Gamma \vdash M_1, M_2 : \tau$ *with an* $\mathbf{x}$ *boundary, we define* $\Gamma \vdash M_1 \lesssim^{\mathbf{x}}_{ter} M_2$ *to hold, when for all contexts* $\vdash C \div \tau$ *built from the syntax of* $\mathbf{x}$*, if* $(C[M_1], \epsilon) \Downarrow_{ter}$ *then* $(C[M_2], \epsilon) \Downarrow_{ter}$*.*

We also consider another way of testing, based on observing whether a program can reach a breakpoint (error point) inside a context. Technically, the breakpoints are represented as occurrences of a special free error variable $err$ : Unit $\to$ Unit. Reaching a breakpoint then corresponds to convergence to a stuck configuration of the form $(K[err()], h)$: we write $(M, h) \Downarrow_{err}$ if there exist $K, h'$ such that $(M, h) \to^* (K[err()], h')$.

**Definition 4 (Contextual Approximation through Error).** *Suppose* $\mathbf{x} \in$ {HOSC, FOSC, HOS, GOS}*. Given* HOSC *terms* $\Gamma \vdash M_1, M_2 : \tau$ *with an* $\mathbf{x}$ *boundary and* $err \notin \mathrm{dom}(\Gamma)$*, we define* $\Gamma \vdash M_1 \lesssim^{\mathbf{x}}_{err} M_2$ *to hold, when for all contexts* $err : \mathrm{Unit} \to \mathrm{Unit} \vdash C \div \tau$ *built from* $\mathbf{x}$*-syntax, if* $(C[M_1], \epsilon) \Downarrow_{err}$ *then* $(C[M_2], \epsilon) \Downarrow_{err}$*.*

For the languages in question, it will turn out that $\lesssim^{\mathbf{x}}_{err}$ is at least as discriminating as $\lesssim^{\mathbf{x}}_{ter}$ for each $\mathbf{x} \in \{\mathrm{HOSC}, \mathrm{GOSC}, \mathrm{HOS}, \mathrm{GOS}\}$, and that they coincide for $\mathbf{x} \in \{\mathrm{HOSC}, \mathrm{GOSC}\}$. We will write $\cong^{\mathbf{x}}_{err}$ and $\cong^{\mathbf{x}}_{ter}$ for the associated equivalence relations.

For higher-order languages with state and control, it is well known that contextual testing can be restricted to evaluation contexts after instantiating the free variables of terms to closed values (the so-called *closed instances of use*, CIU). Let us write $\Sigma, \Gamma' \vdash \gamma : \Gamma$ for substitutions $\gamma$ such that, for any $(x, \sigma_x) \in \Gamma$, the term $\gamma(x)$ is a value satisfying $\Sigma; \Gamma' \vdash \gamma(x) : \sigma_x$. Then $M\{\gamma\}$ stands for the outcome of applying $\gamma$ to $M$.

**Definition 5 (CIU Approximation).** *Let* $\mathbf{x} \in \{\mathrm{HOSC}, \mathrm{GOSC}, \mathrm{HOS}, \mathrm{GOS}\}$ *and let* $\Gamma \vdash M_1, M_2 : \tau$ *be* HOSC *terms with an* $\mathbf{x}$ *boundary.*

- $\Gamma \vdash M_1 \lesssim^{\mathbf{x}(ciu)}_{ter} M_2 : \tau$*, when for all* $\Sigma, h, K, \gamma$*, all built from* $\mathbf{x}$ *syntax, such that* $h : \Sigma$*,* $\Sigma \vdash K \div \tau$*, and* $\Sigma \vdash \gamma : \Gamma$*, we have* $(K[M_1\{\gamma\}], h) \Downarrow_{ter}$ *implies* $(K[M_2\{\gamma\}], h) \Downarrow_{ter}$*.*
- *We write* $\Gamma \vdash M_1 \lesssim^{\mathbf{x}(ciu)}_{err} M_2 : \tau$*, when for all* $\Sigma, h, K, \gamma$*, all built from* $\mathbf{x}$ *syntax, such that* $h : \Sigma; \hat{err}$*,* $\Sigma; \hat{err} \vdash K \div \tau$*, and* $\Sigma; \hat{err} \vdash \gamma : \Gamma$*, we have* $(K[M_1\{\gamma\}], h) \Downarrow_{err}$ *implies* $(K[M_2\{\gamma\}], h) \Downarrow_{err}$*, where* $err \notin \mathrm{dom}(\Gamma)$ *and* $\hat{err}$ *stands for* $err : \mathrm{Unit} \to \mathrm{Unit}$*.*

Results stating that "CIU tests suffice" are referred to as CIU lemmas. A general framework for obtaining such results for higher-order languages with effects was developed in [10,33]. The results stated therein are for termination-based testing, i.e. $\Downarrow_{ter}$, but adapting them to $\Downarrow_{err}$ is not problematic.

**Lemma 1 (CIU Lemma).** *Let* $\mathbf{x} \in \{\mathrm{HOSC}, \mathrm{GOSC}, \mathrm{HOS}, \mathrm{GOS}\}$ *and* $\mathbf{y} \in \{ter, err\}$*. Then we have* $\Gamma \vdash M_1 \lesssim^{\mathbf{x}}_{\mathbf{y}} M_2$ *iff* $\Gamma \vdash M_1 \lesssim^{\mathbf{x}(ciu)}_{\mathbf{y}} M_2$.

The preorders $\lesssim^{\mathbf{x}}_{err}$ will be the central object of study in the paper. Among others, we shall provide their alternative characterizations using trace semantics.The characterizations will apply to a class of terms that we call *cr-free.*

**Definition 6.** *A* HOSC *term* $\Gamma \vdash M : \tau$ *is* **cr-free** *if it does not contain occurrences of* $\mathrm{cont}_\sigma\, K$ *and locations, and its boundary types are* cont- *and* ref*-free.*

We stress that the boundary restriction applies to $\Gamma$ and $\tau$ only, and subterms of $M$ may well contain arbitrary HOSC types and occurrences of $\mathrm{ref}_\sigma$, $\mathrm{call/cc}_\sigma$, $\mathrm{throw}_\sigma$ for any $\sigma$. The majority of HOSC/GOSC/HOS/GOS examples studied in the literature, e.g. [28,4,8], are actually cr-free. We will revisit some of them as Examples 6, 7, 10. The fact that cr-free terms may not contain subterms $\mathrm{cont}_\tau\, K$ or $\ell$ is not really a restriction, as $\mathrm{cont}_\tau\, K$ and $\ell$ being more of a run-time construct than a feature meant to be used directly by programmers. Finally, we note that the boundary of a cr-free term is an $\mathbf{x}$ boundary for any $\mathbf{x} \in \{\mathrm{HOSC}, \mathrm{GOSC}, \mathrm{HOS}, \mathrm{GOS}\}$. Thus, we can consider approximation between cr-terms for any $\mathbf{x}$ from the range, i.e. the notions $\lesssim^{\mathbf{x}}_{err}$, $\lesssim^{\mathbf{x}}_{ter}$ are all applicable. Consequently, cr-free terms provide a common setting in which the discriminating power of HOSC, GOSC, HOS and GOS contexts can be compared. We discuss the scope for extending our results outside of the cr-free fragment, and for richer type systems, in Section 7.

## 3 HOSC[HOSC]

Recall that $\lesssim^{\mathrm{HOSC}}_{err}$ concerns testing HOSC terms with HOSC contexts. Accordingly, we call this case HOSC[HOSC]. For $\mathrm{cont}_{\sigma}(K)$-free terms, we show that $\lesssim^{\mathrm{HOSC}}_{err}$ and $\lesssim^{\mathrm{HOSC}}_{ter}$ coincide, which follows from the lemma below.

**Lemma 2.** *Let* $\Gamma \vdash M_1, M_2$ *be* HOSC *terms not containing any occurrences of* $\mathrm{cont}_{\tau}(K)$*.*

1. $\Gamma \vdash M_1 \lesssim^{\mathbf{x}}_{err} M_2$ *implies* $\Gamma \vdash M_1 \lesssim^{\mathbf{x}}_{ter} M_2$*, for* $\mathbf{x} \in \{\mathrm{HOSC}, \mathrm{GOSC}, \mathrm{HOS}, \mathrm{GOS}\}$*.*
2. $\Gamma \vdash M_1 \lesssim^{\mathbf{x}}_{ter} M_2$ *implies* $\Gamma \vdash M_1 \lesssim^{\mathbf{x}}_{err} M_2$*, for* $\mathbf{x} \in \{\mathrm{HOSC}, \mathrm{GOSC}\}$*.*

In what follows, after introducing several preliminary notions, we shall design a labelled transition system (LTS) whose traces will turn out to capture contextual interactions involved in testing cr-free terms according to $\lesssim^{\mathrm{HOSC}}_{err}$. This will enable us to capture $\lesssim^{\mathrm{HOSC}}_{err}$ via trace inclusion. Actions of the LTS will refer to functions and continuations in a symbolic way, using typed names.

### 3.1 Names and abstract values

**Definition 7.** *Let* $\mathrm{FNames} = \biguplus_{\sigma,\sigma'} \mathrm{FNames}_{\sigma\to\sigma'}$ *be the set of* **function names***, partitioned into mutually disjoint countably infinite sets* $\mathrm{FNames}_{\sigma\to\sigma'}$*. We will use* $f, g$ *to range over* FNames *and write* $f : \sigma \to \sigma'$ *for* $f \in \mathrm{FNames}_{\sigma\to\sigma'}$*.*

*Analogously, let* $\mathrm{CNames} = \biguplus_\sigma \mathrm{CNames}_\sigma$ *be the set of* **continuation names**. *We will use* $c, d$ *to range over* CNames, *and write* $c : \sigma$ *for* $c \in \mathrm{CNames}_\sigma$. *Note that the constants represent continuations, so the "real" type of* $c$ *is* cont $\sigma$, *but we write* $c : \sigma$ *for the sake of brevity. We assume that* $\mathrm{CNames}, \mathrm{FNames}$ *are disjoint and let* $\mathrm{Names} = \mathrm{FNames} \uplus \mathrm{CNames}$. *Elements of* Names *will be weaved into various constructions in the paper, e.g. terms, heaps, etc. We will then write* $\nu(X)$ *to refer to the set of names used in some entity* $X$.

Because of the shape of boundary types in cr-free terms and, in particular, the presence of product types, the values that will be exchanged between the context and the program take the form of tuples consisting of (), integers, booleans and functions. To describe such scenarios, we introduce the notion of ***abstract values***, which are patterns that match such values. Abstract values are generated by the grammar

$$A, B \triangleq () \mid \mathbf{tt} \mid \mathbf{ff} \mid \widehat{n} \mid f \mid \langle A, B \rangle$$

with the proviso that, in any abstract value, a name may occur at most once. As function names are intrinsically typed, we can assign types to abstract values in the obvious way, writing $A : \tau$.

#### 3.2 Actions and traces

Our LTS will be based on four kinds of actions, listed below. Each action will be equipped with a ***polarity***, which is either Player (P) or Opponent (O). P-actions describing interaction steps made by a tested term, while O-actions involve the context.

- ***Player Answer*** (PA) $\bar{c}(A)$, where $c : \sigma$ and $A : \sigma$. This action corresponds to the term sending an abstract value $A$ through a continuation name $c$.
- ***Player Question*** (PQ) $\bar{f}(A, c)$, where $f : \sigma \to \sigma'$, $A : \sigma$ and $c : \sigma'$. Here, an abstract value $A$ and a continuation name $c$ are sent by the term through a function name $f$.
- ***Opponent Answer*** (OA) $c(A)$, $c : \sigma$ then $A : \sigma$. In this case, an abstract value $A$ is received from the environment via the continuation name $c$.
- ***Opponent Question*** (OQ) $f(A, c)$, where $f : \sigma \to \sigma'$, $A : \sigma$ and $c : \sigma'$. Finally, this action corresponds to receiving an abstract value $A$ and a continuation name $c$ from the environment through a function name $f$.

In what follows, $\mathbf{a}$ is used to range over actions. We will say that a name is ***introduced*** by an action $\mathbf{a}$ if it is sent or received in $\mathbf{a}$. If $\mathbf{a}$ is an O-action (resp. P-action), we say that the name was introduced by O (resp. P). An action $\mathbf{a}$ is ***justified*** by another action $\mathbf{a}'$ if the name that $\mathbf{a}$ uses to communicate, i.e. $f$ in questions ($\bar{f}(A, c)$, $f(A, c)$) and $c$ in answers ($\bar{c}(A)$, $c(A)$), has been introduced by $\mathbf{a}'$.

We will work with sequences of actions of a very special shape, specified below. The definition assumes two given sets of names, $N_P$ and $N_O$, which represent names that have already been introduced by P and O respectively.

**Definition 8.** *Let* $N_O, N_P \subseteq$ Names. *An* $(N_O, N_P)$-**trace** *is a sequence* $t$ *of actions such that:*

- *the actions alternate between Player and Opponent actions;*
- *no name is introduced twice;*
- *names from* $N_O, N_P$ *need no introduction;*
- *if an action* $\mathbf{a}$ *uses a name to communicate then*
  - $\mathbf{a} = \bar{f}(A, c)$ $(f \in N_O)$ *or* $\mathbf{a} = \bar{c}(A)$ $(c \in N_O)$ *or* $\mathbf{a} = f(A, c)$ $(f \in N_P)$ *or* $\mathbf{a} = c(A)$ $(c \in N_P)$ *or*
  - *the name has been introduced by an earlier action* $\mathbf{a}'$ *of opposite polarity.*

Note that, due to the shape of actions, a continuation name can only be introduced/justified by a question. Moreover, because names are never introduced twice, if $\mathbf{a}'$ justifies $\mathbf{a}$ then $\mathbf{a}'$ is uniquely determined in a given trace. Readers familiar with game semantics will recognize that traces are very similar to alternating justified sequences except that traces need not be started by O.

*Example 1.* Let $(N_O, N_P) = (\{c\}, \emptyset)$ where $c : \tau = ((\text{Unit} \to \text{Unit}) \to \text{Unit}) \times (\text{Unit} \to \text{Int})$. Then the following sequence is an $(N_O, N_P)$-trace:

$$\mathtt{t}_1 = \bar{c}(\langle g_1, g_2 \rangle)\ \ g_1(f_1, c_1)\ \ \bar{f_1}((), c_2)\ \ c_2(())\ \ \bar{c_1}(())\ \ c_2(())\ \ \bar{c_1}(())\ \ g_2((), c_3)\ \ \bar{c_3}(2)$$

where $g_1 : (\text{Unit} \to \text{Unit}) \to \text{Unit}$, $g_2 : \text{Unit} \to \text{Int}$, $f_1 : \text{Unit} \to \text{Unit}$, $c_1, c_2 :$ Unit, $c_3 :$ Int.

#### 3.3 Extended syntax and reduction

We extend the definition of HOSC presented in Figure 2 to take into account these names. We refine the operational reduction using continuation names to keep track of the toplevel continuation. We list all the changes below.

- Function names are added to the syntax as *constants*. Since they are meant to represent values, they are also considered to be syntactic values in the extended language.
$$\frac{f \in \text{FNames}_{\sigma \to \sigma'}}{\Sigma; \Gamma \vdash f : \sigma \to \sigma'}$$
- Continuation names are *not* terms on their own. Instead, they are built into the syntax via a new construct $\text{cont}_\sigma\,(K, c)$, subject to the following typing rule.
$$\frac{\Sigma; \Gamma \vdash K : \sigma \to \sigma' \quad c \in \text{CNames}_{\sigma'}}{\Sigma; \Gamma \vdash \text{cont}_\sigma\,(K, c) : \text{cont}\ \sigma}$$
  $\text{cont}_\sigma\,(K, c)$ is a staged continuation that first evaluates terms inside $K$ and, if this produces a value, the value is passed to $c$. This operational meaning will be implemented through a suitable reduction rule, to be discussed next. $\text{cont}_\sigma\,(K, c)$ is also regarded as a value. Note that we remove the old construct $\text{cont}_\sigma\,K$ from the extended syntax.

– The operational semantics $\rightarrow$ underpinning the LTS is based on triples $(M, c, h)$ such that $\Sigma; \Gamma \vdash M : \sigma$, $c \in \mathrm{CNames}_\sigma$ and $h : \Sigma$. The continuation name $c$ is used to represent the surrounding context, which is left abstract. The previous operational rules $\rightarrow$ are embedded into the new reduction $\rightarrow$ using the rule below.

$$\frac{(M, h) \rightarrow (M', h')}{(M, c, h) \rightarrow (M', c, h')}$$

The two reduction rules related to continuations, previously used to define $\rightarrow$, are *not* included. Instead we use the following rules, which take advantage of the extended syntax.

$$(K[\mathrm{call/cc}_\tau(x.M)], c, h) \rightarrow (K[M\{\mathrm{cont}_\tau\,(K, c)/x\}], c, h)$$
$$(K[\mathrm{throw}_\tau \text{ V to } \mathrm{cont}_\tau\,(K', c')], c, h) \rightarrow (K'[V], c', h)$$

#### 3.4 Configurations

We write Vals for the extended set of syntactic values, i.e. FNames $\subseteq$ Vals. Let ECtxs stand for the set of extended evaluation contexts, defined as $K$ in Figure 1 taking the extended definition of values into account. Before defining the transition relation of our LTS, we discuss the shape of configurations, providing intuitions behind each component.

***Passive configurations*** take the form $\langle \gamma, \xi, \phi, h \rangle$ and are meant to represent stages at which the environment is to make a move.

– $\gamma : (\mathrm{FNames} \rightharpoonup \mathrm{Vals}) \uplus (\mathrm{CNames} \rightharpoonup \mathrm{ECtxs})$ is a finite map. It will play the role of an environment that relates function names communicated to the environment (i.e. those introduced by P) to syntactic values, and continuation names introduced by P to evaluation contexts.
– $\xi : (\mathrm{CNames} \rightharpoonup \mathrm{CNames})$ is a finite map. It complements the role of $\gamma$ for continuation names and indicates the continuation to which the outcome of applying $\gamma(c)$ should be passed.
– $\phi \subseteq$ Names. The set $\phi$ will be used to collect all the names used in the interaction, regardless of which participant introduced them. Following our description above, those introduced by O will correspond to $\phi \setminus \mathrm{dom}(\gamma)$.

The components satisfy healthiness conditions, implied by their role in the system. Let $\Sigma = \mathrm{dom}(h)$.

– If $f : \mathrm{dom}(\gamma) \cap \mathrm{FNames}_{\sigma \rightarrow \sigma'}$ then $\gamma(f)$ is a value such that $\Sigma \vdash \gamma(f) : \sigma \rightarrow \sigma'$.
– $\mathrm{dom}(\xi) = \mathrm{dom}(\gamma) \cap \mathrm{CNames}$.
– If $c : \mathrm{dom}(\gamma) \cap \mathrm{CNames}_\sigma$ and $\Sigma \vdash \gamma(c) : \sigma \rightarrow \sigma'$ then $\xi(c) \in \mathrm{CNames}_{\sigma'}$.
– Finally, names introduced by the environment and communicated to the program may end up in the environments and the heap: $\nu(\mathrm{img}(\gamma)), \nu(\mathrm{img}(\xi))$, $\nu(\mathrm{img}(h)) \subseteq \phi \setminus \mathrm{dom}(\gamma)$.

***Active configurations*** take the form $\langle M, c, \gamma, \xi, \phi, h \rangle$ and represent interaction steps of the term. The $\gamma, \xi, \phi, h$ components have already been described above. For $M$ and $c$, given $\Sigma = \mathrm{dom}(h)$, we will have $\Sigma; \emptyset \vdash M : \sigma$, $c \in \mathrm{CNames}_\sigma$ and $\nu(M) \cup \{c\} \subseteq \phi \setminus \mathrm{dom}(\gamma)$.

#### 3.5 Transitions

Observe that any closed value $V$ of a cont- and ref-free type $\sigma$ can be decomposed into an abstract value $A$ (pattern) and the corresponding substitution $\gamma$ (matching). The set of all such decompositions, written $\mathbf{AVal}_\sigma(V)$, is defined below. Given a value $V$ of a (cr-free) type $\sigma$, $\mathbf{AVal}_\sigma(V)$ contains all pairs $(A, \gamma)$ such that $A$ is an abstract value and $\gamma : \nu(A) \to \mathrm{Vals}$ is a substitution such that $A\{\gamma\} = V$. More concretely,

$$\begin{array}{ll}
\mathbf{AVal}_\sigma(V) & \triangleq \{(V, \emptyset)\} \quad \text{for } \sigma \in \{\mathrm{Unit}, \mathrm{Bool}, \mathrm{Int}\} \\
\mathbf{AVal}_{\sigma\to\sigma'}(V) & \triangleq \{(f, [f \mapsto V]) \mid f \in \mathrm{FNames}_{\sigma\to\sigma'}\} \\
\mathbf{AVal}_{\sigma\times\sigma'}(\langle U, V\rangle) & \triangleq \{(\langle A_1, A_2\rangle, \gamma_1 \cdot \gamma_2) \mid \\
& \qquad (A_1, \gamma_1) \in \mathbf{AVal}_\sigma(U),\ (A_2, \gamma_2) \in \mathbf{AVal}_{\sigma'}(V)\}
\end{array}$$

Note that, by writing $\cdot$, we mean to implicitly require that the function domains be disjoint. Similarly, when writing $\uplus$, we stipulate that the argument sets be disjoint.

*Example 2.* Let $\sigma = (\mathrm{Int} \to \mathrm{Bool}) \times (\mathrm{Int} \times (\mathrm{Unit} \to \mathrm{Int}))$ and $V \equiv \langle \lambda x^{\mathrm{Int}}.x \neq 1, \langle 2, \lambda x^{\mathrm{Unit}}.3\rangle\rangle$. Then $\mathbf{AVal}_\sigma(V)$ equals

$$\{(\langle f, \langle 2, g\rangle\rangle, [f \mapsto (\lambda x^{\mathrm{Int}}.x \neq 1)] \cdot [g \mapsto (\lambda x^{\mathrm{Unit}}.3)]) \mid \\ f \in \mathrm{FNames}_{\mathrm{Int}\to\mathrm{Unit}},\ g \in \mathrm{FNames}_{\mathrm{Unit}\to\mathrm{Int}}\}.$$

Finally, we present the transitions of what we call the HOSC[HOSC] LTS in Figure 3.

*Example 3.* Below we analyse the (PQ) rule in more detail.

$$\langle K[fV], c, \gamma, \xi, \phi, h\rangle \xrightarrow{\bar{f}(A,c')} \langle \gamma \cdot \gamma' \cdot [c' \mapsto K], \xi \cdot [c' \mapsto c], \phi \uplus \nu(A) \uplus \{c'\}, h\rangle \\ \text{when } f : \sigma \to \sigma',\ (A, \gamma') \in \mathbf{AVal}_\sigma(V) \text{ and } c' : \sigma'$$

The use of $\uplus$ in $\phi \uplus \nu(A) \uplus \{c'\}$ is meant to highlight the requirement that the names introduced in $\bar{f}(A, c')$, i.e. $\nu(A) \cup \{c'\}$, should be fresh and disjoint from $\phi$. Moreover, note how $\gamma$ and $\xi$ are updated. In general, $\gamma, \xi, h$ are updated during P-actions.

**Definition 9.** *Given two configurations* $\mathbf{C}, \mathbf{C}'$, *we write* $\mathbf{C} \overset{\mathbf{a}}{\Rightarrow} \mathbf{C}'$ *if* $\mathbf{C} \xrightarrow{\tau}{}^{*} \mathbf{C}'' \xrightarrow{\mathbf{a}} \mathbf{C}'$, *with* $\xrightarrow{\tau}{}^{*}$ *representing multiple (possibly none)* $\tau$*-actions. This notation is extended to sequences of actions: given* $\mathtt{t} = \mathbf{a}_1 \ldots \mathbf{a}_n$, *we write* $\mathbf{C} \overset{\mathtt{t}}{\Rightarrow} \mathbf{C}'$, *if there exist* $\mathbf{C}_1, \ldots, \mathbf{C}_{n-1}$ *such that* $\mathbf{C} \overset{\mathbf{a}_1}{\Longrightarrow} \mathbf{C}_1 \cdots \mathbf{C}_{n-1} \overset{\mathbf{a}_n}{\Longrightarrow} \mathbf{C}'$. *We define* $\mathbf{Tr}_{\mathrm{HOSC}}(\mathbf{C}) = \{\mathtt{t} \mid \textit{there exists } \mathbf{C}' \textit{ such that } \mathbf{C} \overset{\mathtt{t}}{\Rightarrow} \mathbf{C}'\}$.

**Lemma 3.** *Suppose* $\mathbf{C} = \langle \gamma, \xi, \phi, h\rangle$ *or* $\mathbf{C} = \langle M, c, \gamma, \xi, \phi, h\rangle$ *are configurations. Then elements of* $\mathbf{Tr}_{\mathrm{HOSC}}(\mathbf{C})$ *are* $(\phi \setminus \mathrm{dom}(\gamma), \mathrm{dom}(\gamma))$*-traces.*

$$\begin{array}{l|lll}
(P\tau) & \langle M, c, \gamma, \xi, \phi, h\rangle & \xrightarrow{\tau} & \langle N, c', \gamma, \xi, \phi, h'\rangle \\
& \multicolumn{3}{l}{\text{when } (M, c, h) \rightarrow (N, c', h')} \\
(PA) & \langle V, c, \gamma, \xi, \phi, h\rangle & \xrightarrow{\bar{c}(A)} & \langle \gamma \cdot \gamma', \xi, \phi \uplus \nu(A), h\rangle \\
& \multicolumn{3}{l}{\text{when } c : \sigma,\ (A, \gamma') \in \mathbf{AVal}_\sigma(V)} \\
(PQ) & \langle K[fV], c, \gamma, \xi, \phi, h\rangle & \xrightarrow{\bar{f}(A,c')} & \langle \gamma \cdot \gamma' \cdot [c' \mapsto K], \xi \cdot [c' \mapsto c], \phi \uplus \nu(A) \uplus \{c'\}, h\rangle \\
& \multicolumn{3}{l}{\text{when } f : \sigma \rightarrow \sigma',\ (A, \gamma') \in \mathbf{AVal}_\sigma(V),\ c' : \sigma'} \\
(OA) & \langle \gamma, \xi, \phi, h\rangle & \xrightarrow{c(A)} & \langle K[A], c', \gamma, \xi, \phi \uplus \nu(A), h\rangle \\
& \multicolumn{3}{l}{\text{when } c : \sigma,\ A : \sigma,\ \gamma(c) = K,\ \xi(c) = c'} \\
(OQ) & \langle \gamma, \xi, \phi, h\rangle & \xrightarrow{f(A,c)} & \langle VA, c, \gamma, \xi, \phi \uplus \nu(A) \uplus \{c\}, h\rangle \\
& \multicolumn{3}{l}{\text{when } f : \sigma \rightarrow \sigma',\ A : \sigma,\ c : \sigma',\ \gamma(f) = V}
\end{array}$$

NB $c : \sigma$ stands for $c \in \text{CNames}_\sigma$.

**Fig. 3.** HOSC[HOSC] LTS

| $M_1^{cwl}$ | $M_2^{cwl}$ |
|---|---|
| $M_1^{cwl}$ : let x = ref 0 in<br>let b = ref **ff** in<br>⟨λf. if ¬(!b) then<br>b := **tt**; f(); x :=!x + 1;<br>b := **ff**;<br>else (), λ_ : Unit.!x⟩ | $M_2^{cwl}$ : let x = ref 0 in<br>let b = ref **ff** in<br>⟨λf. if ¬(!b) then<br>b := **tt**; let n =!x in f(); x := n + 1;<br>b := **ff**;<br>else () , λ_ : Unit.!x⟩ |

**Fig. 4.** Callback-with-lock Example [4]

*Example 4.* In Figure 5, we show that the trace from Example 1 is generated by the configuration $\mathbf{C} \triangleq \langle M_1^{cwl}, c, \emptyset, \emptyset, \{c\}, \emptyset\rangle$, where $M_1^{cwl}$ is given in Figure 4. We write inc $\triangleq \lambda f.\text{if } \neg(!\ell_b)\ (\ell_b := \mathbf{tt}; f(); \ell_x :=!\ell_x + 1; \ell_b := \mathbf{ff})\ ()$, get $\triangleq \lambda\_.!\ell_x$ and $c : ((\text{Unit} \rightarrow \text{Unit}) \rightarrow \text{Unit}) \times (\text{Unit} \rightarrow \text{Int})$. It is interesting to notice that in this interaction, Opponent uses the continuation $N$ twice, incrementing the counter $x$ by two. The second time, it does it without having to call inc again, but rather by using the continuation name $c_2$.

*Remark 2.* Due to the freedom of name choice, note that $\mathbf{Tr}_{\text{HOSC}}(\mathbf{C})$ is closed under type-preserving renamings that preserve names from $\mathbf{C}$.

#### 3.6 Correctness and full abstraction

We define two kinds of special configurations that will play an important role in spelling out correctness results for the HOSC[HOSC] LTS. Let $\Gamma = \{x_1 : \sigma_1, \cdots, x_k : \sigma_k\}$. A map $\rho$ from $\{x_1, \cdots, x_k\}$ to the set of abstract values will be called a ***Γ-assignment*** provided, for all $1 \leq i \neq j \leq k$, we have $\rho(x_i) : \sigma_i$ and $\nu(\rho(x_i)) \cap \nu(\rho(x_j)) = \emptyset$.

$$
\begin{array}{ll}
\mathbf{C} = \langle M_1^{cwl}, c, \emptyset, \emptyset, \{c\}, \emptyset\rangle & \\
\xrightarrow{\tau^*} \langle\langle \mathrm{inc}, \mathrm{get}\rangle, c, \emptyset, \emptyset, \{c\}, [\ell_b \mapsto \mathbf{ff}, \ell_x \mapsto 0]\rangle & \\
\xrightarrow{\bar{c}(\langle g_1, g_2\rangle)} \langle \gamma_1, \emptyset, \{c, g_1, g_2\}, [\ell_b \mapsto \mathbf{ff}, \ell_x \mapsto 0]\rangle & \text{with } \gamma_1 = [g_1 \mapsto \mathrm{inc}, g_2 \mapsto \mathrm{get}], \\
\xrightarrow{g_1(f_1, c_1)} \langle \mathrm{inc} f_1, c_1, \gamma_1, \emptyset, \phi_2, [\ell_b \mapsto \mathbf{ff}, \ell_x \mapsto 0]\rangle & \text{with } \phi_2 = \{c, g_1, g_2, f_1, c_1\} \\
\xrightarrow{\tau^*} \langle f_1(); N, c_1, \gamma_1, \emptyset, \phi_2, [\ell_b \mapsto \mathbf{tt}, \ell_x \mapsto 0]\rangle & \text{with } N = \ell_x := !\ell_x + 1; \ell_b := \mathbf{ff} \\
\xrightarrow{\bar{f_1}((), c_2)} \langle \gamma_2, \xi, \phi_3, [\ell_b \mapsto \mathbf{tt}, \ell_x \mapsto 0]\rangle & \text{with } \gamma_2 = \gamma_1 \cdot [c_2 \mapsto \bullet; N], \\
\xrightarrow{c_2(())} \langle (); N, c_1, \gamma_2, \xi, \phi_3, [\ell_b \mapsto \mathbf{tt}, \ell_x \mapsto 0]\rangle & \quad \xi = [c_2 \mapsto c_1] \text{ and } \phi_3 = \phi_2 \uplus \{c_2\} \\
\xrightarrow{\tau^*} \langle (), c_1, \gamma_2, \xi, \phi_3, [\ell_b \mapsto \mathbf{ff}, \ell_x \mapsto 1]\rangle & \\
\xrightarrow{\bar{c_1}(())} \langle \gamma_2, \xi, \phi_3, [\ell_b \mapsto \mathbf{ff}, \ell_x \mapsto 1]\rangle & \\
\xrightarrow{c_2(())} \langle (); N, c_1, \gamma_2, \xi, \phi_3, [\ell_b \mapsto \mathbf{ff}, \ell_x \mapsto 1]\rangle & \\
\xrightarrow{\tau^*} \langle (), c_1, \gamma_2, \xi, \phi_3, [\ell_b \mapsto \mathbf{ff}, \ell_x \mapsto 2]\rangle & \\
\xrightarrow{\bar{c_1}(())} \langle \gamma_2, \xi, \phi_3, [\ell_b \mapsto \mathbf{ff}, \ell_x \mapsto 2]\rangle & \\
\xrightarrow{g_2((), c_3)} \langle \mathrm{get}(), c_3, \gamma_2, \xi, \phi_4, [\ell_b \mapsto \mathbf{ff}, \ell_x \mapsto 2]\rangle & \text{with } \phi_4 = \phi_3 \uplus \{c_3\} \\
\xrightarrow{\tau^*} \langle 2, c_3, \gamma_2, \xi, \phi_4, [\ell_b \mapsto \mathbf{ff}, \ell_x \mapsto 2]\rangle & \\
\xrightarrow{\bar{c_3}(2)} \langle \gamma_2, \xi, \phi_4, [\ell_b \mapsto \mathbf{ff}, \ell_x \mapsto 2]\rangle &
\end{array}
$$

**Fig. 5.** Trace derivation in the HOSC[HOSC] LTS

**Definition 10 (Program configuration).** *Given a $\Gamma$-assignment $\rho$, a cr-free HOSC term $\Gamma \vdash M : \tau$ and $c : \tau$, we define the active configuration $\mathsf{C}_M^{\rho,c}$ by $\mathsf{C}_M^{\rho,c} = \langle M\{\rho\}, c, \emptyset, \emptyset, \nu(\rho) \cup \{c\}, \emptyset\rangle$.*

Note that traces from $\mathbf{Tr}_{\mathrm{HOSC}}(\mathsf{C}_M^{\rho,c})$ will be $(\nu(\rho) \cup \{c\}, \emptyset)$-traces.

**Definition 11.** *The* HOSC[HOSC] **trace semantics** *of a cr-free HOSC term $\Gamma \vdash M : \tau$ is defined to be*

$$\mathbf{Tr}_{\mathrm{HOSC}}(\Gamma \vdash M : \tau) = \{((\rho, c), t) \mid \rho \textit{ is a } \Gamma\textit{-assignment}, \, c : \tau, \, t \in \mathbf{Tr}_{\mathrm{HOSC}}(\mathsf{C}_M^{\rho,c})\}.$$

*Example 5.* Recall the term $\vdash M_1^{cwl} : \tau$ from Example 4, the trace $\mathtt{t}_1$ and the configuration $\mathbf{C}$ such that $\mathtt{t}_1 \in \mathbf{Tr}_{\mathrm{HOSC}}(\mathbf{C})$. Because $M_1^{cwl}$ is closed ($\Gamma = \emptyset$), the only $\Gamma$-assignment is the empty map $\emptyset$. Thus, $\mathbf{C} = \mathsf{C}_{M_1^{cwl}}^{\emptyset,c}$, so $((\emptyset, c), \mathtt{t}_1) \in \mathbf{Tr}_{\mathrm{HOSC}}(\vdash M_1^{cwl} : \tau)$.

Having defined active configurations associated with terms, we now define passive configurations associated with contexts. Let us fix $\diamond \in \mathrm{FNames}_{\mathrm{Unit} \to \mathrm{Unit}}$ and, for each $\sigma$, a continuation name $\circ_\sigma \in \mathrm{CNames}_\sigma$. Let $\circ = \bigcup_\sigma \{\circ_\sigma\}$. Intuitively, the names $\diamond$ will correspond to $\Downarrow_{err}$ and $\circ_\sigma$ to $\Downarrow_{ter}$.

Recall that $\hat{err}$ stands for $err$ : Unit $\to$ Unit. Given a heap $h : \Sigma; \hat{err}$, an evaluation context $\Sigma; \hat{err} \vdash K : \tau \to \tau'$ and a substitution $\Sigma; \hat{err} \vdash \gamma : \Gamma$ (as in the definition of $\lesssim_{err}^{\mathrm{HOSC}(ciu)}$), let us replace every occurrence of $\mathrm{cont}_\sigma\, K'$ inside $h, K, \gamma$ with $\mathrm{cont}_\sigma\, (K', \circ_{\sigma'})$, if $K'$ has type $\sigma \to \sigma'$. Moreover, let us replace every occurrence of the variable $err$ with the function name $\diamond$. This is done to adjust $h, K, \gamma$ to the extended syntax of the LTS: the upgraded versions are called $h_\circ, \gamma_\circ, K_\circ$.

Next we define the set $\mathbf{AVal}_\Gamma(\gamma)$ of all disjoint decompositions of values from $\gamma_\circ$ into abstract values and the corresponding matchings. Recall that $\Gamma = \{x_1 : \sigma_1, \cdots, x_k : \sigma_k\}$. Below $\vec{A_i}$ stands for $(A_1, \cdots, A_k)$, and $\vec{\gamma_i}$ for $(\gamma_1, \cdots, \gamma_k)$.

$$\mathbf{AVal}_\Gamma(\gamma) = \{ \quad (\vec{A_i}, \vec{\gamma_i}) \mid (A_i, \gamma_i) \in \mathbf{AVal}_{\sigma_i}(\gamma_\circ(x_i)),\ i = 1, \cdots, k; \\ \nu(A_1), \cdots, \nu(A_k) \text{ mutually disjoint and without } \diamond \quad \}$$

**Definition 12 (Context configuration).** *Given $\Sigma$, $h : \Sigma; \hat{err}$, $\Sigma; \hat{err} \vdash K : \tau \to \tau'$, $\Sigma; \hat{err} \vdash \gamma : \Gamma$, $(\vec{A_i}, \vec{\gamma_i}) \in \mathbf{AVal}_\Gamma(\gamma)$ and $c : \tau$ ($c \notin \circ$), the corresponding configuration $\mathsf{C}^{\vec{\gamma_i},c}_{h,K,\gamma}$ is defined by*

$$\mathsf{C}^{\vec{\gamma_i},c}_{h,K,\gamma} = \langle \biguplus_{i=1}^{k} \gamma_i \uplus \{c \mapsto K_\circ\}, \{c \mapsto \circ_{\tau'}\}, \biguplus_{i=1}^{k} \nu(A_i) \uplus \{c\} \uplus \circ \uplus \{\diamond\}, h_\circ \rangle.$$

Intuitively, the names $\nu(A_i)$ correspond to calling function values extracted from $\gamma$, whereas $c$ corresponds to $K$. Note that traces in $\mathbf{Tr}_{\mathrm{HOSC}}(\mathsf{C}^{\vec{\gamma_i},c}_{h,K,\gamma})$ will be $(\circ \uplus \{\diamond\}, \biguplus_{i=1}^{k} \nu(A_i) \uplus \{c\})$-traces.

In preparation for the next result, we introduce the following shorthands.

– Given a $(N_O, N_P)$-trace $t$, we write $t^\perp$ for the $(N_P, N_O)$-trace obtained by changing the polarity of each name: $f(A, c')$ becomes $\bar{f}(A, c')$ (and vice versa) and $c(A)$ becomes $\bar{c}(A)$ (and vice versa).
– Given $(\vec{A_i}, \vec{\gamma_i}) \in \mathbf{AVal}_\Gamma(\gamma)$, we define a $\Gamma$-assignment $\rho_{\vec{A_i}}$ by $\rho_{\vec{A_i}}(x_i) = A_i$. Note that $\nu(\rho_{\vec{A_i}}) = \biguplus_{i=1}^{k} \mathrm{dom}(\gamma_i)$.

**Lemma 4 (Correctness).** *Let $\Gamma \vdash M : \tau$ be a cr-free* HOSC *term, let $\Sigma, h, K, \gamma$ be as above, $(\vec{A_i}, \vec{\gamma_i}) \in \mathbf{AVal}_\Gamma(\gamma)$, and $c : \tau$ ($c \notin \circ$). Then*

– *$(K[M\{\gamma\}], h) \Downarrow_{err}$ iff there exist $t, c'$ such that $t \in \mathbf{Tr}_{\mathrm{HOSC}}(\mathsf{C}^{\rho_{\vec{A_i}},c}_{M})$ and $t^\perp\, \bar{\diamond}((), c') \in \mathbf{Tr}_{\mathrm{HOSC}}(\mathsf{C}^{\vec{\gamma_i},c}_{h,K,\gamma})$.*
– *$(K[M\{\gamma\}], h) \Downarrow_{ter}$ iff there exist $t, A, \sigma$ such that $t \in \mathbf{Tr}_{\mathrm{HOSC}}(\mathsf{C}^{\rho_{\vec{A_i}},c}_{M})$ and $t^\perp\, \bar{\circ}_\sigma(A) \in \mathbf{Tr}_{\mathrm{HOSC}}(\mathsf{C}^{\vec{\gamma_i},c}_{h,K,\gamma})$.*

*Moreover, $t$ satisfies $\nu(t) \cap (\circ \cup \{\diamond\}) = \emptyset$.*

Intuitively, the lemma above confirms that the potential of a term to converge is determined by its traces. Accordingly, we have:

**Theorem 1 (Soundness).** *For any cr-free* HOSC *terms $\Gamma \vdash M_1, M_2$, if $\mathbf{Tr}_{\mathrm{HOSC}}(\Gamma \vdash M_1) \subseteq \mathbf{Tr}_{\mathrm{HOSC}}(\Gamma \vdash M_2)$ then $\Gamma \vdash M_1 \lesssim^{\mathrm{HOSC}(ciu)}_{err} M_2$.*

To prove the converse, we need to know that every odd-length trace generated by a term actually participates in a contextual interaction. This will follow from the lemma below. Note that $\Downarrow_{err}$ relies on even-length traces from the context (Lemma 4).

**Lemma 5 (Definability).** *Suppose* $\phi \uplus \{\diamond\} \subseteq$ FNames *and* $t$ *is an even-length* $(\circ \uplus \{\diamond\}, \phi \uplus \{c\})$*-trace starting with an O-action. There exists a passive configuration* $\mathbf{C}$ *such that the even-length traces* $\mathbf{Tr}_{\mathrm{HOSC}}(\mathbf{C})$ *are exactly the even-length prefixes of* $t$ *(along with all renamings that preserve types and* $\phi \uplus \{c\} \uplus \circ \uplus \{\diamond\}$*, cf. Remark 2). Moreover,* $\mathbf{C} = \langle \gamma_\circ \cdot [c \mapsto K_\circ], \{c \mapsto \circ_{\tau'}\}, \phi \uplus \{c\} \uplus \circ \uplus \{\diamond\}, h_\circ \rangle$*, where* $h, K, \gamma$ *are built from* HOSC *syntax.*

*Proof (Sketch).* The basic idea is to use references in order to record all continuation and function names introduced by the environment. For continuations, the use of call/cc$_\tau$ is essential. Once stored in the heap, the names can be accessed by terms when needed in P-actions. The availability of throw and references to all O-continuations means that arbitrary answer actions can be scheduled when needed.

**Theorem 2 (Completeness).** *For any cr-free* HOSC *terms* $\Gamma \vdash M_1, M_2$*,* $\Gamma \vdash M_1 \lesssim_{err}^{\mathrm{HOSC}(ciu)} M_2$ *implies* $\mathbf{Tr}_{\mathrm{HOSC}}(\Gamma \vdash M_1) \subseteq \mathbf{Tr}_{\mathrm{HOSC}}(\Gamma \vdash M_2)$*.*

Theorems 1, 2 (along with Lemmas 1, 2) imply the following full abstraction results.

**Corollary 1 (HOSC Full Abstraction).** *Suppose* $\Gamma \vdash M_1, M_2$ *are cr-free* HOSC *terms. Then* $\mathbf{Tr}_{\mathrm{HOSC}}(\Gamma \vdash M_1) \subseteq \mathbf{Tr}_{\mathrm{HOSC}}(\Gamma \vdash M_2)$ *iff* $\Gamma \vdash M_1 \lesssim_{err}^{\mathrm{HOSC}} M_2$ *iff* $\Gamma \vdash M_1 \lesssim_{ter}^{\mathrm{HOSC}} M_2$*.*

*Example 6 (Callback with lock [4]).* Recall the term $\vdash M_1^{cwl} : ((\mathrm{Unit} \to \mathrm{Unit}) \to \mathrm{Unit}) \times (\mathrm{Unit} \to \mathrm{Int})$ from Example 4, given in Figure 4. We had $\mathtt{t}_1 = \bar{c}(\langle g_1, g_2 \rangle)\; g_1(f_1, c_1)\; \bar{f}_1((), c_2)\; c_2(())\; \bar{c}_1(())\; c_2(())\; \bar{c}_1(())\; g_2((), c_3)\; \bar{c}_3(2) \in \mathbf{Tr}_{\mathrm{HOSC}}(\mathsf{C}^{\emptyset,c}_{M_1^{cwl}})$.

Define $\mathtt{t}_2$ to be $\mathtt{t}_1$ except that its last action $\bar{c}_3(2)$ is replaced with $\bar{c}_3(1)$. Observe that $\mathtt{t}_1 \in \mathbf{Tr}_{\mathrm{HOSC}}(\mathsf{C}^{\emptyset,c}_{M_1^{cwl}}) \setminus \mathbf{Tr}_{\mathrm{HOSC}}(\mathsf{C}^{\emptyset,c}_{M_2^{cwl}})$ and $\mathtt{t}_2 \in \mathbf{Tr}_{\mathrm{HOSC}}(\mathsf{C}^{\emptyset,c}_{M_2^{cwl}}) \setminus \mathbf{Tr}_{\mathrm{HOSC}}(\mathsf{C}^{\emptyset,c}_{M_1^{cwl}})$, i.e. by the Corollary above the terms are incomparable wrt $\lesssim_{err}^{\mathrm{HOSC}}$. However, they are equivalent wrt $\lesssim_{err}^{\mathbf{x}}$ for $\mathbf{x} \in \{\mathrm{GOSC}, \mathrm{HOS}, \mathrm{GOS}\}$ [8].

The above Corollary also provides a handle to reason about equivalence via trace equivalence. Sometimes this can be done directly on the LTS, especially when $\gamma$ can be kept bounded.

*Example 7 (Counter [28]).* For $i \in \{1, 2\}$, consider the terms $\vdash M_i : (\mathrm{Unit} \to \mathrm{Unit}) \times (\mathrm{Unit} \to \mathrm{Int})$ given by $M_i \equiv \mathrm{let}\, x = \mathrm{ref}\, 0\, \mathrm{in}\, \langle \mathsf{inc}_i, \mathsf{get}_i \rangle$, where $\mathsf{inc}_1 \equiv (\lambda y.x := !x + 1)$, $\mathsf{inc}_2 \equiv (\lambda y.x := !x - 1)$, $\mathsf{get}_1 \equiv \lambda z.!x$, $\mathsf{get}_2 \equiv \lambda z.-!x$. In this case, $\mathbf{Tr}_{\mathrm{HOSC}}(\mathsf{C}^{\emptyset,c}_{M_i})$ contains (prefixes of) traces of the form $\bar{c}(\langle g, h \rangle)\, t$, where $t$ is built from segments of two kinds: either $g((), c_i)\; \bar{c}_i(())$ or $h((), c_i')\; \bar{c}_i'(n)$, where the $c_i$s and $c_i'$s are pairwise different. Moreover, in the latter case, $n$ must be equal to the number of preceding actions of the form $g((), c_i)$. For this example, trace equality could be established by induction on the length of trace. Consequently, $M_1 \cong_{err}^{\mathrm{HOSC}} M_2$.

## 4 GOSC[HOSC]

Recall that GOSC is the fragment of HOSC in which general storage is restricted to values of *ground* type, i.e. arithmetic/boolean constants, the associated reference names, references to those names and so on. In what follows, we are going to provide characterizations of $\lesssim_{err}^{\mathrm{GOSC}}$ via trace inclusion. Recall that, by Lemma 2, $\lesssim_{err}^{\mathrm{GOSC}} = \lesssim_{ter}^{\mathrm{GOSC}}$. Note that we work in an asymmetric setting with terms belonging to HOSC being more powerful than contexts.

We start off by identifying several technical consequences of the restriction to GOSC syntax. First we observe that GOSC internal reductions never contribute extra names.

**Lemma 6.** *Suppose* $(M, c, h) \to (M', c', h')$*, where* $M$ *is a* GOSC *term and* $h$ *is a* GOSC *heap. Then* $\nu(M) \cup \{c\} \supseteq \nu(M') \cup \{c'\}$*.*

*Proof.* By case analysis. All defining rules for $\to$, with the exception of the $(K[!\ell], h) \to (K[h(\ell)], h)$ rule, are easily seen to satisfy the Lemma (no function or continuation names are added). However, if the heap is restricted to storing elements of type $\iota$ (as in GOSC) then $h(\ell)$ will never contain a name, so the Lemma follows.

The lemma has interesting consequences for the shape of traces generated by the context configurations $\mathsf{C}_{h,K,\gamma}^{\vec{\gamma_i},c}$ if they are built from GOSC syntax. Recall that P-actions have the form $\bar{f}(A, c')$ or $\bar{c}(A)$, where $f, c$ are names introduced by O. It turns out that when $h, K, \gamma$ are restricted to GOSC, more can be said about the origin of the names in traces generated by $\mathsf{C}_{h,K,\gamma}^{\vec{\gamma_i},c}$: they will turn out to come from a restricted set of names introduced by O, which we identify below. The definition below is based on following the justification structure of a trace – recall that one action is said to justify another if the former introduces a name that is used for communication in the latter.

**Definition 13.** *Suppose* $\phi \uplus \{\diamond\} \subseteq \mathrm{FNames}$ *and* $c \in \mathrm{CNames}$*. Let* $t$ *be an odd-length* $(\circ \uplus \{\diamond\}, \phi \uplus \{c\})$*-trace starting with an O-action. The set* $\mathsf{Vis}_P(t)$ *of P-visible names of* $t$ *is defined as follows.*

$$\begin{array}{rcll}
\mathsf{Vis}_P(t\ \ c'(A')) &=& \{\diamond\} \cup \circ \cup \nu(A') & c' = c \\
\mathsf{Vis}_P(t\ \ \bar{f}''(A'', c')\ \ t'\ \ c'(A')) &=& \mathsf{Vis}_P(t) \cup \nu(A') & c' \neq c \\
\mathsf{Vis}_P(t\ \ f'(A', c')) &=& \{\diamond\} \cup \circ \cup \nu(A') \cup \{c'\} & f' \in \phi \\
\mathsf{Vis}_P(t\ \ \bar{f}''(A'', c'')\ \ t'\ \ f'(A', c')) &=& \mathsf{Vis}_P(t) \cup \nu(A') \cup \{c'\} & f' \in \nu(A'') \\
\mathsf{Vis}_P(t\ \ \bar{c}''(A'')\ \ t'\ \ f'(A', c')) &=& \mathsf{Vis}_P(t) \cup \nu(A') \cup \{c'\} & f' \in \nu(A'')
\end{array}$$

Note that, in the inductive cases, the definition follows links between names introduced by P and the point of their introduction, names introduced in-between are ignored. Here readers familiar with game semantics will notice similarity to the notion of P-view [12].

Next we specify a property of traces that will turn out to be satisfied by configurations corresponding to GOSC contexts.

**Definition 14.** *Suppose* $\phi \uplus \{\diamond\} \subseteq$ FNames *and* $c \in$ CNames. *Let* $t$ *be a* $(\circ \uplus \{\diamond\}, \phi \uplus \{c\})$*-trace starting with an O-action.* $t$ *is called* **P-visible** *if*

- *for any even-length prefix* $t'\ \bar{f}(A,c)$ *of* $t$*, we have* $f \in \mathsf{Vis}_P(t')$*,*
- *for any even-length prefix* $t'\ \bar{c}(A)$ *of* $t$*, we have* $c \in \mathsf{Vis}_P(t')$*.*

**Lemma 7.** *Consider* $\mathbf{C} = \mathsf{C}_{h,K,\gamma}^{\vec{\gamma_i},c}$*, where* $h, K, \gamma$ *are from* GOSC *and* $(\vec{A_i}, \vec{\gamma_i}) \in \mathbf{AVal}_\Gamma(\gamma)$*. Then all traces in* $\mathbf{Tr}_{\mathrm{HOSC}}(\mathbf{C})$ *are P-visible.*

The Lemma above shows that contextual interactions with GOSC contexts rely on restricted traces. We shall now modify the HOSC[HOSC] LTS to capture the restriction. Note that, from the perspective of the term, the above constraint is a constraint on the use of names by O (context), so we need to talk about O-available names instead. This dual notion is defined below.

**Definition 15.** *Suppose* $\phi \subseteq$ FNames *and* $c \in$ CNames. *Let* $t$ *be a* $(\phi \uplus \{c\}, \emptyset)$*-trace of odd length. The set* $\mathsf{Vis}_O(t)$ *of* **O-visible names** *of* $t$ *is defined as follows.*

$$
\begin{array}{rcll}
\mathsf{Vis}_O(t\ \ \bar{c'}(A')) &=& \nu(A') & c' = c \\
\mathsf{Vis}_O(t\ \ f''(A'',c')\ \ t'\ \ \bar{c'}(A')) &=& \mathsf{Vis}_O(t) \cup \nu(A') & c' \neq c \\
\mathsf{Vis}_O(t\ \ \bar{f'}(A',c')) &=& \nu(A') \cup \{c'\} & f' \in \phi \\
\mathsf{Vis}_O(t\ \ f''(A'',c'')\ \ t'\ \ \bar{f'}(A',c')) &=& \mathsf{Vis}_O(t) \cup \nu(A') \cup \{c'\} & f' \in \nu(A'') \\
\mathsf{Vis}_O(t\ \ c''(A'')\ \ t'\ \ \bar{f'}(A',c')) &=& \mathsf{Vis}_O(t) \cup \nu(A') \cup \{c'\} & f' \in \nu(A'')
\end{array}
$$

*Analogously, a* $(\phi \uplus \{c\}, \emptyset)$*-trace* $t$ *is* **O-visible** *if, for any even-length prefix* $t'\ f(A,c)$ *of* $t$*, we have* $f \in \mathsf{Vis}_O(t')$ *and, for any even-length prefix* $t'\ c(A)$ *of* $t$*, we have* $c \in \mathsf{Vis}_O(t')$*.*

*Example 8.* Recall the trace

$$
\mathtt{t}_1 = \bar{c}(\langle g_1, g_2\rangle)\ \ g_1(f_1,c_1)\ \ \bar{f_1}((),c_2)\ \ c_2(())\ \ \bar{c_1}(())\ \ c_2(())\ \ \bar{c_1}(())\ \ g_2((),c_3)\ \ \bar{c_3}(2)
$$

from previous examples. Observe that

$$
\begin{array}{rcl}
\mathsf{Vis}_O(\bar{c}(\langle g_1, g_2\rangle)\ g_1(f_1,c_1)\ \bar{f_1}((),c_2)) &=& \{g_1, g_2, c_2\} \\
\mathsf{Vis}_O(\bar{c}(\langle g_1, g_2\rangle)\ g_1(f_1,c_1)\ \bar{f_1}((),c_2)\ c_2(())\ \bar{c_1}(())) &=& \{g_1, g_2\}
\end{array}
$$

Consequently, the first use of $c_2(())$ in $\mathtt{t}_1$ does not violate O-visibility, but the second one does.

In Figure 6, we present a new LTS, called the GOSC[HOSC] LTS, which will turn out to capture $\lesssim_{err}^{\mathrm{GOSC}}$ through trace inclusion. It is obtained from the HOSC[HOSC] LTS by restricting O-actions to those that rely on O-visible names. Technically, this is done by enriching configurations with an additional component $\mathcal{F}$, which maintains historical information about O-available names immediately before each O-action. After each P-action, $\mathcal{F}$ is accessed to calculate the current set $\mathcal{V}$ of O-available names according to the definition of O-availability and only O-actions compatible with O-availability are allowed to proceed (due

$$
\begin{array}{l|l}
(P\tau) & \langle M, c, \gamma, \xi, \phi, h, \mathcal{F}\rangle \xrightarrow{\tau} \langle N, c', \gamma, \xi, \phi, h', \mathcal{F}\rangle \\
& \text{when } (M, c, h) \to (N, c', h') \\
(PA) & \langle V, c, \gamma, \xi, \phi, h, \mathcal{F}\rangle \xrightarrow{\bar{c}(A)} \langle \gamma \cdot \gamma', \xi, \phi \uplus \nu(A), h, \mathcal{F}, \mathcal{F}(c) \uplus \nu(A)\rangle \\
& \text{when } c : \sigma \text{ and } (A, \gamma') \in \mathbf{AVal}_\sigma(V) \\
(PQ) & \langle K[fV], c, \gamma, \xi, \phi, h, \mathcal{F}\rangle \xrightarrow{\bar{f}(A,c')} \\
& \qquad \langle \gamma \cdot \gamma' \cdot [c' \mapsto K], \xi \cdot [c' \mapsto c], \phi \uplus \phi', h, \mathcal{F}, \mathcal{F}(f) \uplus \phi'\rangle \\
& \text{when } f : \sigma \to \sigma',\ (A, \gamma') \in \mathbf{AVal}_\sigma(V),\ c' : \sigma' \text{ and } \phi' = \nu(A) \uplus \{c'\} \\
(OA) & \langle \gamma, \xi, \phi, h, \mathcal{F}, \mathcal{V}\rangle \xrightarrow{c(A)} \langle K[A], c', \gamma, \xi, \phi \uplus \nu(A), h, \mathcal{F} \cdot [\nu(A) \mapsto \mathcal{V}]\rangle \\
& \text{when } c \in \mathcal{V},\ c : \sigma,\ A : \sigma,\ \gamma(c) = K,\ \xi(c) = c' \\
(OQ) & \langle \gamma, \xi, \phi, h, \mathcal{F}, \mathcal{V}\rangle \xrightarrow{f(A,c)} \langle VA, c, \gamma, \xi, \phi \uplus \phi', h, \mathcal{F} \cdot [\phi' \mapsto \mathcal{V}]\rangle \\
& \text{when } f \in \mathcal{V},\ f : \sigma \to \sigma',\ A : \sigma,\ c : \sigma',\ \gamma(f) = V \text{ and } \phi' = \nu(A) \uplus \{c\}
\end{array}
$$

Given $N \subseteq$ Names, $[N \mapsto \mathcal{V}]$ stands for the map $[n \mapsto \mathcal{V} \mid n \in N]$.

**Fig. 6.** GOSC[HOSC] LTS

to the $f \in \mathcal{V}$, $c \in \mathcal{V}$ side conditions). We write $\mathbf{Tr}_{\text{GOSC}}(\mathbf{C})$ for the set of traces generated from $\mathbf{C}$ in the GOSC[HOSC] LTS.

Recall that, given a $\Gamma$-assignment $\rho$, term $\Gamma \vdash M : \tau$ and $c \in \text{CNames}_\tau$, the active configuration $\mathsf{C}_M^{\rho,c}$ was defined by $\mathsf{C}_M^{\rho,c} = \langle M\{\rho\}, c, \emptyset, \emptyset, \nu(\rho) \cup \{c\}, \emptyset\rangle$. We need to upgrade it to the LTS by initializing the new component to the empty map: $\mathsf{C}_{M,vis}^{\rho,c} = \langle M\{\rho\}, c, \emptyset, \emptyset, \nu(\rho) \cup \{c\}, \emptyset, \emptyset\rangle$.

**Definition 16.** *The* GOSC[HOSC] **trace semantics** *of a cr-free HOSC term* $\Gamma \vdash M : \tau$ *is defined by* $\mathbf{Tr}_{\text{GOSC}}(\Gamma \vdash M : \tau) = \{((\rho, c), t) \mid \rho$ *is a* $\Gamma$*-assignment,* $c : \tau,\ t \in \mathbf{Tr}_{\text{GOSC}}(\mathsf{C}_{M,vis}^{\rho,c})\}$.

By construction, it follows that

**Lemma 8.** $t \in \mathbf{Tr}_{\text{GOSC}}(\mathsf{C}_{M,vis}^{\rho,c})$ *iff* $t \in \mathbf{Tr}_{\text{HOSC}}(\mathsf{C}_M^{\rho,c})$ *and* $t$ *is O-visible.*

Noting that the witness trace $t$ from Lemma 4 is O-visible iff $t^\perp\, \bar{\diamond}((), c')$ is P-visible, we can conclude that, for GOSC, the traces relevant to $\Downarrow_{err}$ are O-visible, which yields:

**Theorem 3 (Soundness).** *For any cr-free* HOSC *terms* $\Gamma \vdash M_1,\ M_2$*, if* $\mathbf{Tr}_{\text{GOSC}}(\Gamma \vdash M_1) \subseteq \mathbf{Tr}_{\text{GOSC}}(\Gamma \vdash M_2)$ *then* $\Gamma \vdash M_1 \lesssim_{err}^{\text{GOSC}(ciu)} M_2$.

To prove the converse, we need a new definability result. This time we are only allowed to use GOSC syntax, but the target is also more modest: we are only aiming to capture P-visible traces.

**Lemma 9 (Definability).** *Suppose* $\phi \uplus \{\diamond\} \subseteq$ FNames *and* $t$ *is an even-length* P-visible $(\circ \uplus \{\diamond\}, \phi \uplus \{c\})$*-trace starting with an O-action. There exists a passive configuration* $\mathbf{C}$ *such that the even-length traces in* $\mathbf{Tr}_{\text{HOSC}}(\mathbf{C})$ *are exactly the even-length prefixes of* $t$ *(along with all renamings that preserve types and* $\phi \uplus \{c\} \uplus \circ \uplus \{\diamond\}$*). Moreover,* $\mathbf{C} = \langle \gamma_\circ \cdot [c \mapsto K_\circ], \{c \mapsto \circ_{\tau'}\}, \phi \uplus \{c\} \uplus \circ \uplus \{\diamond\}, h_\circ\rangle$*, where* $h, K, \gamma$ *are built from* GOSC *syntax.*

*Proof (Sketch).* This time we cannot rely on references to recall on demand all continuation and function names introduced by the environment. However, because $t$ is P-visible, it turns the uses of the names can be captured through variable bindings ($\lambda x. \cdots$ for function and $\mathrm{call/cc}_\tau(x. \dots)$ for continuation names). Using throw, we can then force an arbitrary answer action, as long as it uses a P-available name. To select the right action at each step, we branch on the value of a single global reference of type ref Int that keeps track of the number of steps simulated so far.

Completeness now follows because, for a potential O-visible witness $t$ from Lemma 4, one can create a corresponding context by invoking the Definability result for $t^\perp\, \bar{\diamond}((), c')$. It is crucial that the addition of $\bar{\diamond}((), c')$ does not break P-visibility ($\diamond$ is P-visible).

**Theorem 4 (Completeness).** *For any cr-free* HOSC *terms* $\Gamma \vdash M_1, M_2$*, if* $\Gamma \vdash M_1 \lesssim_{err}^{\mathrm{GOSC}(ciu)} M_2$ *then* $\mathbf{Tr}_{\mathrm{GOSC}}(\Gamma \vdash M_1) \subseteq \mathbf{Tr}_{\mathrm{GOSC}}(\Gamma \vdash M_2)$*.*

Altogether, Theorems 3, 4 (along with Lemma 1) imply the following result.

**Corollary 2** (GOSC **Full Abstraction).** *Suppose* $\Gamma \vdash M_1, M_2$ *are cr-free* HOSC *terms.* $\mathbf{Tr}_{\mathrm{GOSC}}(\Gamma \vdash M_1) \subseteq \mathbf{Tr}_{\mathrm{GOSC}}(\Gamma \vdash M_2)$ *iff* $\Gamma \vdash M_1 \lesssim_{err}^{\mathrm{GOSC}(ciu)} M_2$ *iff* $\Gamma \vdash M_1 \lesssim_{err}^{\mathrm{GOSC}} M_2$*.*

*Example 9.* In the *Callback with lock* example (Example 6), we exhibited traces $\mathtt{t}_1, \mathtt{t}_2$ that separated $M_1^{cwl}$ and $M_2^{cwl}$ with respect to $\lesssim_{err}^{\mathrm{HOSC}}$. Example 8 shows that neither trace is O-visible, i.e. they do not belong to $\mathbf{Tr}_{\mathrm{GOSC}}(\Gamma \vdash M_1)$ or $\mathbf{Tr}_{\mathrm{GOSC}}(\Gamma \vdash M_2)$. Thus, the two traces cannot be used to separate $M_1^{cwl}, M_2^{cwl}$ with respect to $\lesssim_{err}^{\mathrm{GOSC}}$. As already mentioned, this is in fact impossible: we have $\vdash M_1^{cwl} \cong_{err}^{\mathrm{GOSC}} M_2^{cwl}$.

*Example 10 (Well-bracketed state change [4]).* Consider the following two terms

$$M_1^{wbsc} \triangleq \mathrm{let}\, x = \mathrm{ref}\, 0\, \mathrm{in}\, \lambda f.(x := 0; f(); x := 1; f(); !x)$$
$$M_2^{wbsc} \triangleq \lambda f.(f(); f(); 1).$$

of type $\tau = (\mathrm{Unit} \to \mathrm{Unit}) \to \mathrm{Int}$, let

$$\mathtt{t}_3 = \bar{c}(g)\;\; g(f_1, c_1)\;\; \bar{f_1}((), c_2)\;\; c_2(())\;\; \bar{f_1}((), c_3)\;\; g(f_2, c_4)\;\; \bar{f_2}((), c_5)\;\; c_3(())\;\; \bar{c_1}(0)$$

and let $\mathtt{t}_4$ be obtained from $\mathtt{t}_3$ by changing 0 in the last action to 1. One can check that both traces are O-visible: in particular, the action $c_3(())$ is not a violation because

$$\mathsf{Vis}_O(\bar{c}(g)\;\; g(f_1, c_1)\;\; \bar{f_1}((), c_2)\;\; c_2(())\;\; \bar{f_1}((), c_3)\;\; g(f_2, c_4)\;\; \bar{f_2}((), c_5)) = \{g, c_3, c_5\}.$$

Moreover, $\mathtt{t}_3 \in \mathbf{Tr}_{\mathrm{GOSC}}(\mathsf{C}^{\emptyset,c}_{M_1^{wbsc}}) \setminus \mathbf{Tr}_{\mathrm{GOSC}}(\mathsf{C}^{\emptyset,c}_{M_2^{wbsc}})$ and $\mathtt{t}_4 \in \mathbf{Tr}_{\mathrm{GOSC}}(\mathsf{C}^{\emptyset,c}_{M_2^{wbsc}}) \setminus \mathbf{Tr}_{\mathrm{GOSC}}(\mathsf{C}^{\emptyset,c}_{M_1^{wbsc}})$. By the Corollary above, we can conclude that $M_1^{wbsc}, M_2^{wbsc}$ are incomparable wrt $\lesssim_{err}^{\mathrm{GOSC}}$. However, they turn out to be $\cong_{err}^{\mathrm{HOS}}$- and $\cong_{err}^{\mathrm{GOS}}$-equivalent.

## 5 HOS[HOSC]

Recall that HOS is the fragment of HOSC that does not feature continuation types and the associated syntax. In what follows we are going to provide alternative characterisations of $\lesssim_{err}^{\text{HOS}}$ and $\lesssim_{ter}^{\text{HOS}}$ in terms of trace inclusion and complete trace inclusion respectively.

We start off by identifying several technical consequences of the restriction to HOS syntax. First we observe that HOS internal reductions never change the associated continuation name.

**Lemma 10.** *If* $(M, c, h) \to (M', c', h')$*,* $M$ *is a* HOS *term and* $h$ *is a* HOS *heap then* $c = c'$*.*

*Proof.* The only rule that could change $c$ is the rule for throw, but it is not part of HOS.

The lemma has a bearing on the shape of traces generated by the (passive) configurations $\mathsf{C}_{h,K,\gamma}^{\vec{\gamma_i},c}$ corresponding to HOS contexts. In the presence of throw and storage for continuations, it was possible for P to play answers involving arbitrary continuation names introduced by O. By Lemma 10, in HOS this will be restricted to the continuation name of the current configuration, which will restrict the shape of possible traces. Below we identify the continuation name $top_P(t)$ that becomes the relevant name after trace $t$. If the last move was an O-question then the continuation name introduced by that move will become that name. Otherwise, we track a chain of answers and questions, similarly to the definition of P-visibility.

Observe that, because $h, K, \gamma$ are from HOS, $\mathsf{C}_{h,K,\gamma}^{\vec{\gamma_i},c}$ will generate $(\{\circ_{\tau'}, \diamond\}, \phi \uplus \{c\})$-traces, where $\tau'$ is the result type of $K$, because $h_\circ = h, K_\circ = K, \gamma_\circ = \gamma$.

**Definition 17.** *Suppose* $\phi \uplus \{\diamond\} \subseteq$ FNames *and* $c \in$ CNames*. Let* $t$ *be a* $(\{\circ_{\tau'}, \diamond\}, \phi \uplus \{c\})$*-trace of odd length starting with an O-action. The continuation name* $top_P(t)$ *is defined as follows.*

$$\begin{aligned} top_P(t\ c(A)) &= \circ_{\tau'} \\ top_P(t_1\ \bar{f}(A'', c')\ t_2\ c'(A')) &= top_P(t_1) \\ top_P(t\ f(A', c')) &= c' \end{aligned}$$

*We say that a* $(\{\circ_{\tau'} \cup \{\diamond\}, \phi \uplus \{c\})$*-trace* $t$ *starting with an O-action is* **P-bracketed** *if, for any prefix* $t'\ \bar{c'}(A)$ *of* $t$ *(i.e. any prefix ending with a P-answer), we have* $c' = top_P(t')$*.*

**Lemma 11.** *Consider* $\mathbf{C} = \mathsf{C}_{h,K,\gamma}^{\vec{\gamma_i},c}$*, where* $h, K, \gamma$ *are from* HOS *and* $(\vec{A_i}, \vec{\gamma_i}) \in$ $\mathbf{AVal}_\Gamma(\gamma)$*. Then all traces in* $\mathbf{Tr}_{\text{HOSC}}(\mathbf{C})$ *are P-bracketed.*

The Lemma above characterizes the restrictive nature of contextual interactions with HOS contexts. Next we shall constrain the HOSC[HOSC] LTS accordingly to capture the restriction. Note that, from the point of view of the term, the above-mentioned constraint concerns the use of continuation names by O (the context), so we need to talk about O-bracketing instead. This dual notion of "a top name for O" is specified below.

$$
\begin{array}{l|lcl}
(P\tau) & \langle M, c, \gamma, \xi, \phi, h\rangle & \xrightarrow{\tau} & \langle N, c', \gamma, \xi, \phi, h'\rangle \\
& \text{when } (M, c, h) \to (N, c', h') & & \\
(PA) & \langle V, c, \gamma, \xi, \phi, h\rangle & \xrightarrow{\bar{c}(A)} & \langle \gamma \cdot \gamma', \xi, \phi \uplus \nu(A), h, c'\rangle \\
& \text{when } c : \sigma,\ (A, \gamma') \in \mathbf{AVal}_\sigma(V),\ \xi(c) = c' & & \\
(PQ) & \langle K[fV], c, \gamma, \xi, \phi, h\rangle & \xrightarrow{\bar{f}(A,c')} & \langle \gamma \cdot \gamma' \cdot [c' \mapsto K], \xi \cdot [c' \mapsto c], \phi \uplus \nu(A) \uplus \{c'\}, h, c'\rangle \\
& \text{when } f : \sigma \to \sigma',\ (A, \gamma') \in \mathbf{AVal}_\sigma(V),\ c' : \sigma' & & \\
(OA) & \langle \gamma, \xi, \phi, h, c''\rangle & \xrightarrow{c(A)} & \langle K[A], c', \gamma, \xi, \phi \uplus \nu(A), h\rangle \\
& \text{when } c = c'',\ c : \sigma,\ A : \sigma,\ \gamma(c) = K,\ \xi(c) = c' & & \\
(OQ) & \langle \gamma, \xi, \phi, h, c''\rangle & \xrightarrow{f(A,c)} & \langle VA, c, \gamma, \xi \cdot [c \mapsto c''], \phi \uplus \nu(A) \uplus \{c\}, h\rangle \\
& \text{when } f : \sigma \to \sigma',\ A : \sigma,\ c : \sigma',\ \gamma(f) = V & &
\end{array}
$$

**Fig. 7.** HOS[HOSC] LTS

**Definition 18.** *Suppose* $\phi \subseteq \text{FNames}$ *and* $c \in \text{CNames}$. *Let* $t$ *be a* $(\phi \uplus \{c\}, \emptyset)$*-trace of odd length. The continuation name* $top_O(t)$ *is defined as follows. In the first case, the value is* $\bot$ *(representing "none"), because* $c$ *is the top continuation passed by the environment to the term (if it gets answered there is nothing left to answer).*

$$
\begin{aligned}
top_O(t\ \bar{c}(A)) &= \bot \\
top_O(t_1\ f(A'', c')\ t_2\ \bar{c'}(A')) &= top_O(t_1) \\
top_O(t\ \bar{f}(A', c')) &= c'
\end{aligned}
$$

*We say that a* $(\phi \uplus \{c\}, \emptyset)$*-trace* $t$ *is* **O-bracketed** *if, for any prefix* $t'\ c'(A)$ *of* $t$ *(i.e. any prefix ending with an O-answer), we have* $c' = top_O(t')$.

In Figure 7, we present a new LTS, called the HOS[HOSC] LTS, which will turn out to capture $\lesssim^{\text{HOS}}_{err}$. It is obtained from the HOSC[HOSC] LTS by restricting O-actions to those that satisfy O-bracketing. Technically, this is done by enriching passive configurations with a component for storing the current value of $top_O(t)$. In order to maintain this information, we need to know which continuation will become the top one if P plays an answer. This can be done with a map that maps continuations introduced by O to other continuations. Because its flavour is similar to $\xi$ (which is a map from continuations introduced by P) we integrate this information into $\xi$. The $c = c''$ side condition then enforces O-bracketing. We shall write $\mathbf{Tr}_{\text{HOS}}(\mathbf{C})$ for the set of traces generated from $\mathbf{C}$ in the HOS[HOSC] LTS.

Recall that, given a $\Gamma$-assignment $\rho$, term $\Gamma \vdash M : \tau$ and $c : \tau$, the active configuration $\mathsf{C}^{\rho,c}_M$ was defined by $\mathsf{C}^{\rho,c}_M = \langle M\{\rho\}, c, \emptyset, \emptyset, \nu(\rho) \cup \{c\}, \emptyset\rangle$. We upgrade it to the new LTS by setting $\mathsf{C}^{\rho,c}_{M,bra} = \langle M\{\rho\}, c, \emptyset, [c \mapsto \bot], \nu(\rho) \cup \{c\}, \emptyset, \emptyset\rangle$. This initializes $\xi$ in such a way that, after $\bar{c}(A)$ is played, the extra component will be set to $\bot$, where $\bot$ is a special element not in CNames.

**Definition 19.** *The* HOS[HOSC] **trace semantics** *of a cr-free HOSC term* $\Gamma \vdash M : \tau$ *is defined to be* $\mathbf{Tr}_{\mathrm{HOS}}(\Gamma \vdash M : \tau) = \{((\rho, c), t) \mid \rho$ *is a* $\Gamma$*-assignment,* $c : \tau$, $t \in \mathbf{Tr}_{\mathrm{HOS}}(\mathsf{C}^{\rho;c}_{M,bra})\}$.

By construction, it follows that

**Lemma 12.** $t \in \mathbf{Tr}_{\mathrm{HOS}}(\mathsf{C}^{\rho;c}_{M,bra})$ *iff* $t \in \mathbf{Tr}_{\mathrm{HOSC}}(\mathsf{C}^{\rho;c}_{M})$ *and* $t$ *is O-bracketed.*

Noting that the witness trace $t$ from Lemma 4 is O-bracketed iff $t^{\perp}\,\bar{\diamond}((), c')$ is P-bracketed, we can conclude that, for HOS, the traces relevant to $\Downarrow_{err}$ are O-bracketed, which yields:

**Theorem 5 (Soundness).** *For any cr-free* HOSC *terms* $\Gamma \vdash M_1, M_2$*, if* $\mathbf{Tr}_{\mathrm{HOS}}(\Gamma \vdash M_1) \subseteq \mathbf{Tr}_{\mathrm{HOS}}(\Gamma \vdash M_2)$ *then* $\Gamma \vdash M_1 \lesssim^{\mathrm{HOS}(ciu)}_{err} M_2$.

For the converse, we establish another definability result, this time for a P-bracketed trace.

**Lemma 13 (Definability).** *Suppose* $\phi \uplus \{\diamond\} \subseteq$ FNames *and* $t$ *is an even-length* P-bracketed $(\{\circ_{\tau'}, \diamond\}, \phi \uplus \{c\})$*-trace starting with an O-action. There exists a passive configuration* $\mathbf{C}$ *such that the even-length traces* $\mathbf{Tr}_{\mathrm{HOSC}}(\mathbf{C})$ *are exactly the even-length prefixes of* $t$ *(along with all renamings that preserve types and* $\phi \uplus \{c, \circ_{\tau'}, \diamond\}$*). Moreover,* $\mathbf{C} = \langle \gamma \cdot [c \mapsto K], \{c \mapsto \circ_{\tau'}\}, \phi \uplus \{c, \circ_{\tau'}, \diamond\}, h\rangle$*, where* $h, K, \gamma$ *are built from* HOS *syntax.*

*Proof (Sketch).* Our argument for HOSC is structured in such a way that, for a P-bracketed trace, there is no need for continuations (throwing and continuation capture are not necessary).

Completeness now follows because, for a potential witness trace $t$ from Lemma 4, one can create a corresponding context by invoking the Definability result for $t^{\perp}\,\bar{\diamond}((), c')$. It is crucial that the addition of $\bar{\diamond}((), c')$ does not break P-bracketing (it does not, because the action is a question).

**Theorem 6 (Completeness).** *For any cr-free* HOSC *terms* $\Gamma \vdash M_1, M_2$*, if* $\Gamma \vdash M_1 \lesssim^{\mathrm{HOS}(ciu)}_{err} M_2$ *then* $\mathbf{Tr}_{\mathrm{HOS}}(\Gamma \vdash M_1) \subseteq \mathbf{Tr}_{\mathrm{HOS}}(\Gamma \vdash M_2)$.

Altogether, Theorems 5, 6 (along with Lemma 1) imply the following result.

**Corollary 3** (HOS **Full Abstraction).** *Suppose* $\Gamma \vdash M_1, M_2$ *are cr-free* HOSC *terms. Then* $\mathbf{Tr}_{\mathrm{HOS}}(\Gamma \vdash M_1) \subseteq \mathbf{Tr}_{\mathrm{HOS}}(\Gamma \vdash M_2)$ *iff* $\Gamma \vdash M_1 \lesssim^{\mathrm{HOS}(ciu)}_{err} M_2$ *iff* $\Gamma \vdash M_1 \lesssim^{\mathrm{HOS}}_{err} M_2$.

*Example 11 (Assignment/callback commutation [27]).* For $i \in \{1, 2\}$, let $f$ : Unit $\to$ Unit $\vdash M_i$ : Unit $\to$ Unit be defined by:

$$M_1 \triangleq \mathrm{let}\, n = \mathrm{ref}\,(0)\,\mathrm{in}\,\lambda y^{\mathrm{Unit}}.\mathrm{if}\ (!n > 0)\ ()\ (n := 1; f()),$$
$$M_2 \triangleq \mathrm{let}\, n = \mathrm{ref}\,(0)\,\mathrm{in}\,\lambda y^{\mathrm{Unit}}.\mathrm{if}\ (!n > 0)\ ()\ (f(); n := 1).$$

Operationally, one can see that $f \vdash M_1 \not\lesssim_{err}^{\mathrm{HOS}} M_2$ due to the following HOS context: $\mathrm{let}\, r = \mathrm{ref}\,(\lambda y.y)\,\mathrm{in}\,(\mathrm{let}\, f = \lambda y.(!r)()\,\mathrm{in}\,(r := \bullet; (!r)())); \mathit{err}$. In our framework, this is confirmed by the trace

$$\mathtt{t}_5 \quad = \quad \bar{c}(g) \quad g((), c_1) \quad \bar{f}((), c_2) \quad g((), c_2) \quad \bar{c_2}(()),$$

which is in $\mathbf{Tr}_{\mathrm{HOS}}(\mathsf{C}_{M_1}^{\rho,c}) \setminus \mathbf{Tr}_{\mathrm{HOS}}(\mathsf{C}_{M_2}^{\rho,c})$. On the other hand,

$$\mathtt{t}_6 \quad = \quad \bar{c}(g) \quad g((), c_1) \quad \bar{f}((), c_2) \quad g((), c_2) \quad \bar{f}((), c_3)$$

is in $\mathbf{Tr}_{\mathrm{HOS}}(\mathsf{C}_{M_2}^{\rho,c}) \setminus \mathbf{Tr}_{\mathrm{HOS}}(\mathsf{C}_{M_1}^{\rho,c})$, so the terms are incomparable. Note, however, that both traces break O-visibility: specifically, we have

$$\mathsf{Vis}_O(\bar{c}(g)\ g((), c_1)\ \bar{f}((), c_2)) = \{c_2\},$$

so the $g((), c_2)$ action violates the condition. Consequently, the traces do not preclude $f \vdash M_1 \cong_{err}^{\mathbf{x}} M_2$ for $\mathbf{x} \in \{\mathrm{GOSC}, \mathrm{GOS}\}$.

For $\mathbf{x} \in \{\mathrm{HOSC}, \mathrm{GOSC}\}$, $\lesssim_{err}^{\mathbf{x}}$ and $\lesssim_{ter}^{\mathbf{x}}$ coincide. Intuitively, this is because the presence of continuations in the context makes it possible to make an escape at any point. In contrast, for HOS, the context must run to completion in order to terminate.

At the technical level, one can appreciate the difference when trying to transfer our results for $\lesssim_{err}^{\mathrm{HOS}(ciu)}$ to $\lesssim_{ter}^{\mathrm{HOS}(ciu)}$. Recall that, according to Lemma 4, $\Downarrow_{ter}$ relies on a witness trace $t$ such that the context configuration generates $t^{\perp}\ \bar{\circ}_{\tau'}()$. In HOS, the latter must satisfy P-bracketing, so we need $\mathit{top}_P(t^{\perp}) = \circ_{\tau'}$. Note that this is equivalent to $\mathit{top}_O(t) = \perp$. Consequently, only such traces are relevant to observing $\Downarrow_{ter}$.

We shall call an odd-length O-bracketed $(\phi \uplus \{c\}, \emptyset)$-trace $t$ ***complete*** if $\mathit{top}_O(t) = \perp$. Let us write $\mathbf{Tr}_{\mathrm{HOS}}(\Gamma \vdash M_1) \subseteq_c \mathbf{Tr}_{\mathrm{HOS}}(\Gamma \vdash M_2)$ if we have $((\rho, c), t) \in \mathbf{Tr}_{\mathrm{HOS}}(\Gamma \vdash M_2)$ whenever $((\rho, c), t) \in \mathbf{Tr}_{\mathrm{HOS}}(\Gamma \vdash M_1)$ and $t$ is complete. Following our methodology, one can then show:

**Theorem 7 (**HOS **Full Abstraction for $\lesssim_{ter}^{\mathrm{HOS}}$).** *Suppose $\Gamma \vdash M_1, M_2$ are cr-free* HOSC *terms.* $\mathbf{Tr}_{\mathrm{HOS}}(\Gamma \vdash M_1) \subseteq_c \mathbf{Tr}_{\mathrm{HOS}}(\Gamma \vdash M_2)$ *iff* $\Gamma \vdash M_1 \lesssim_{ter}^{\mathrm{HOS}(ciu)} M_2$ *iff* $\Gamma \vdash M_1 \lesssim_{ter}^{\mathrm{HOS}} M_2$.

*Example 12.* Let $M_1 \equiv \lambda f^{\mathrm{Unit}\to\mathrm{Unit}}.f(); \Omega_{\mathrm{Unit}}$ and $M_2 \equiv \lambda f^{\mathrm{Unit}\to\mathrm{Unit}}.\Omega_{\mathrm{Unit}}$. We will see that $\vdash M_1 \not\lesssim_{err}^{\mathrm{HOS}} M_2$ but $\vdash M_1 \lesssim_{ter}^{\mathrm{HOS}} M_2$. To see this, note that $\mathbf{Tr}_{\mathrm{HOS}}(\mathsf{C}_{M_1}^{\rho,c})$ contains prefixes of $\bar{c}(g)\ g(f, c_1)\ \bar{f}((), c_2)\ c_2(())$, while $\mathbf{Tr}_{\mathrm{HOS}}(\mathsf{C}_{M_2}^{\rho,c})$ only those of $\bar{c}(g)\ g(f, c_1)$. Observe that the only complete trace among them is $\bar{c}(g)$. The trace $t = \bar{c}(g)\ g(f, c_1)\ \bar{f}((), c_2)$ is not complete, because $\mathit{top}_O(t) = c_2$. Consequently, $\mathbf{Tr}_{\mathrm{HOS}}(\Gamma \vdash M_1) \not\subseteq \mathbf{Tr}_{\mathrm{HOS}}(\Gamma \vdash M_2)$ but $\mathbf{Tr}_{\mathrm{HOS}}(\Gamma \vdash M_1) \subseteq_c \mathbf{Tr}_{\mathrm{HOS}}(\Gamma \vdash M_2)$.

The theorem above generalizes the characterisation of contextual equivalence between HOS terms with respect to HOS contexts [23], where trace completeness means both O- and P-bracketing and "all questions must be answered". Our definition of completeness is weaker (O-bracketing + "the top question must be answered"), because it also covers HOSC terms. However, in the presence of both O- and P-bracketing, i.e. for HOS terms, they will coincide.

### 6 GOS[HOSC]

Recall that GOS features ground state only and, technically, is the intersection of GOSC and HOS. Consequently, it follows from the previous sections that GOS contexts yield configurations that satisfy both P-visibility and P-bracketing. For such traces, the definability result for GOSC yields a GOS context. Thus, in a similar fashion to the previous sections, we can conclude that O-visible and O-bracketed traces underpin $\lesssim_{err}^{\mathrm{GOS}}$. To define the GOS LTS we simply combine the restrictions imposed in the previous sections, and define $\mathbf{Tr}_{\mathrm{GOS}}(\Gamma \vdash M)$ analogously. The results on $\lesssim_{ter}^{\mathrm{GOS}}$ from the previous section also carry over to GOS.

**Theorem 8** (GOS **Full Abstraction).** *Suppose* $\Gamma \vdash M_1, M_2$ *are cr-free* HOSC *terms. Then:*

– $\mathbf{Tr}_{\mathrm{GOS}}(\Gamma \vdash M_1) \subseteq \mathbf{Tr}_{\mathrm{GOS}}(\Gamma \vdash M_2)$ *iff* $\Gamma \vdash M_1 \lesssim_{err}^{\mathrm{GOS}(ciu)} M_2$ *iff* $\Gamma \vdash M_1 \lesssim_{err}^{\mathrm{GOS}} M_2$.
– $\mathbf{Tr}_{\mathrm{GOS}}(\Gamma \vdash M_1) \subseteq_c \mathbf{Tr}_{\mathrm{GOS}}(\Gamma \vdash M_2)$ *iff* $\Gamma \vdash M_1 \lesssim_{ter}^{\mathrm{GOS}(ciu)} M_2$ *iff* $\Gamma \vdash M_1 \lesssim_{ter}^{\mathrm{GOS}} M_2$.

### 7 Concluding remarks

*Asymmetry* Our framework is able to deal with asymmetric scenarios, where programs are taken from HOSC, but are tested with contexts from weaker fragments. For example, we can compare the following two HOSC programs, where $f : ((\mathrm{Unit} \to \mathrm{Unit}) \to \mathrm{Unit}) \to \mathrm{Unit}$ is a free identifier.

| | |
|---|---|
| let b = ref **ff** in callcc(y. | callcc(y. |
| f(λg.b := **tt**; g(); throw() to y); | f(λg.g(); throw() to y); |
| if !b then () else div) | div) |

with div representing divergence. The terms happen to be $\cong_{err}^{\mathrm{HOS}}$-equivalent, but not $\cong_{err}^{\mathrm{HOSC}}$-equivalent.

To see this at the intuitive level, we make the following observations.

– Firstly, we observe that, to distinguish the terms, f should use its argument. Otherwise, the value of b will remain equal to **ff**, and the only subterm that distinguishes the terms (‘if !b then () else div’) will play the same role as div in the second term.
– Secondly, if f does use its argument, then b will be set to **tt** in the first program, raising the possibility of distinguishing the terms. However, if we allow HOS contexts only then, since the argument to f was used, it will have to run to completion, before ‘if !b then () else div’ is reached. Consequently, we will encounter ‘throw () to $y$’ earlier and never reach ‘if !b then () else div’. This is represented by the trace

$$\bar{f}(h, c_1) \qquad h(g, c_2) \qquad \bar{g}((), c_3) \qquad c_3(()) \qquad \bar{c}(())$$

This trace is O-bracketed, but not P-bracketed since Player uses throw to answer directly to the initial continuation $c$ rather than $c_2$.

– Finally, if HOSC contexts are allowed, it is possible to reach the subterm 'if !b then () else div' with b set to **tt**. This is represented by the trace

$$\bar{f}(h, c_1) \qquad h(g, c_2) \qquad \bar{g}((), c_3) \qquad c_1(()) \qquad \bar{c}(())$$

This trace is not O-bracketed, because $c_1$ is answered rather than $c_3$, like above. Consequently, the trace witnesses termination of the first term, but the second term would diverge during interaction with the same context.

We plan to explore the opportunities presented by this setting in the future, especially with respect to fully abstract translations, for example, from HOSC to GOS.

*Richer Types* Recall that our full abstraction results are stated for cr-free terms, terms with cont- and ref-free types at the boundary. Here we first discuss how to extend them to more complicated types.

To deal with reference type at the boundary, i.e. location exchange, one needs to generalize the notion of traces, so that they can carry, for each action, a heap representing the values stored in the disclosed part of the heap, as in [23,27]. The extension to sum, recursive and empty types seems conceptually straightforward, by simply extending the definition of abstract values for these types, following the similar notion of ultimate pattern in [24]. The same idea should apply to allow continuation types at the boundary. Operational game semantics for an extension of HOS with polymorphism has been explored in [15].

*Innocence* On the other hand, all of the languages we considered were stateful. In the presence of state, all of the actions that are represented by labels (and their order and frequency) can be observed, because they could generate a side-effect. A natural question to ask whether the techniques could also be used to provide analogous theorems for purely functional computation, i.e. contexts taken from the language PCF. Here, the situation is different. For example, the terms $f : \mathrm{Int} \to \mathrm{Int} \vdash f(0)$ and $f : \mathrm{Int} \to \mathrm{Int} \vdash \text{if } f(0)\ f(0)\ f(0)$ should be equivalent, even though the sets of their traces are incomparable.

It is known [12] that PCF strategies satisfy a uniformity condition called innocence. Unfortunately, restricting our traces to "O-innocent ones" (like we did with O-visibility and O-bracketing) would not deliver the required characterization. Technically, this is due to the fact that, in our arguments, given a single trace (with suitable properties), we can produce a context that induces the given trace and no other traces (except those implied by the definition of a trace). For innocence, this would not be possible due to the uniformity requirement. It will imply that, although we can find a functional context that generates an innocent trace, it might also generate other traces, which then have to be taken into account when considering contextual testing. This branching property makes it difficult to capture equivalence with respect to functional contexts explicitly, e.g. through traces, which is illustrated by the use of the so-called intrinsic quotient in game models of PCF [2,12].

## 8 Related Work

We have presented four operational game models for HOSC, which capture term interaction with contexts built from any of the four sublanguages $\mathbf{x} \in \{$HOSC, GOSC, HOS, GOS$\}$ respectively. The most direct precursor to this work is Laird's trace model for HOS[HOS] [23]. Other frameworks in this spirit include models for objects [18], aspects [16] and system-level code [9]. In [13], Laird's model has been related formally to the denotational game model from [27]. However, in general, it is not yet clear how one can move systematically between the operational and denotational game-based approaches, despite some promising steps reported in [25]. Below we mention other operational techniques for reasoning about contextual equivalence.

In [31], fully abstract Eager-Normal-Form (enf) Bisimulations are presented for an untyped $\lambda$-calculus with store and control, similar to HOSC (but with control represented using the $\lambda\mu$-calculus). The bisimulations are parameterised by worlds to model the evolution of store, and bisimulations on contexts are used to deal with control. Like our approach, they are based on symbolic evaluation of open terms. Typed enf-bisimulations, for a language without store and in control-passing style, have been introduced in [24]. Fully-abstract enf-bisimulations are presented in [7] for a language with state only, corresponding to an untyped version of HOS. Earlier works in this strand include [17,29].

Environmental Bisimulations [19,30,32] have also been introduced for languages with store. They work on closed terms, computing the arguments that contexts can provide to terms using an environment similar to our component $\gamma$. They have also been extended to languages with call/cc [34] and delimited control operators [5,6].

Kripke Logical Relations [28,4,8] have been introduced for languages with state and control. In [8], a characterization of contextual equivalence for each case $\mathbf{x}[\mathbf{x}]$ ($\mathbf{x} \in \{\text{HOSC}, \text{GOSC}, \text{HOS}, \text{GOS}\}$) is given, using techniques called backtracking and public transitions, which exploit the absence of higher-order store and that of control constructs respectively. Importing these techniques in the setting of Kripke Open Bisimulations [14] should allow one to build a bridge between the game-semantics characterizations and Kripke Logical Relations.

Parametric bisimulations [11] have been introduced as an operational technique, merging ideas from Kripke Logical Relations and Environmental Bisimulations. They do not represent functional values coming from the environment using names, but instead use a notion of global and local knowledge to compute these values, reminiscent of the work on environmental bisimulations. The notion of global knowledge depends itself on a notion of evolving world. To our knowledge, no fully abstract Parametric Bisimulations have been presented.

A general theory of applicative [21] and normal-form bisimulations [20] has been developed, with the goal of being modular with respect to the effects considered. While the goal is similar to our work, the papers consider monadic and algebraic presentation of effects, trying particularly to design a general theory for proving soundness and completeness of such bisimulations. These works complement ours, and we would like to explore possible connections.
